# Cerebral oxygen monitoring during out-of-hospital cardiac arrest: A scoping review

**DOI:** 10.1016/j.resplu.2025.101082

**Published:** 2025-09-03

**Authors:** Ingjerd Baugstø, Nora Gjesdal, Sarah Elizabeth King, Sindre Andre Pedersen, Lars Petter Bache-Wiig Bjørnsen, Nils Kristian Skjærvold, Oddvar Uleberg

**Affiliations:** a Faculty of Medicine and Health Sciences, Norwegian University of Science and Technology (NTNU), NO-7006 Trondheim, Norway; b Department of Circulation and Medical Imaging, Norwegian University of Science and Technology (NTNU), NO-7006 Trondheim, Norway; c Library Section for Research Support, Data and Analysis, The Medicine and Health Library, Norwegian University of Science and Technology (NTNU), NO-7006 Trondheim, Norway; d Department of Emergency Medicine and Pre-hospital Services, St. Olav s University Hospital, NO-7006 Trondheim, Norway; e Department of Anesthesia and Intensive Care Medicine, St. Olav’s University Hospital, NO-7006 Trondheim, Norway; f Norwegian Air Ambulance Foundation, Department of Research and Development, NO-0103 Oslo, Norway

**Keywords:** Cardiac arrest, Cerebral oxygenation, Near-infrared spectroscopy, Brain monitoring, Resuscitation, Emergency medicine, Prehospital care

## Abstract

**Background:**

Cerebral tissue oxygen saturation is not routinely monitored during out-of-hospital cardiac arrest (OHCA) but can be assessed using cerebral oxygen monitoring (COM). This non-invasive technique provides real-time measurements of brain oxygenation. The aim of this scoping review (ScR) was to map and describe published studies on the use of COM in OHCA in prehospital and emergency department (ED) settings.

**Methods:**

This review followed the JBI Manual for Evidence Synthesis and the Preferred Reporting Items for Systematic reviews and Meta-Analyses ScR (PRISMA-ScR) checklist. Peer-reviewed primary studies reporting the use of COM in OHCA patients in prehospital and/or ED settings were eligible. MEDLINE, Embase, CINAHL, Cochrane Library, and Web of Science were searched from inception to August 8th, 2025.

**Results:**

A total of 2166 records were screened, and 57 publications representing 51 studies were included. The studied populations were mainly adult males and included patients with non-traumatic causes of arrest. Most studies were small observational cohorts from Japan, conducted in the ED. Near-infrared spectroscopy (NIRS) was the predominant technology used. Studies commonly examined associations between COM values and return of spontaneous circulation (ROSC) or neurological outcomes. Few papers evaluated COM’s impact on clinical decision-making or treatment.

**Conclusion:**

The evidence base is dominated by small, ED-based observational studies with limited geographical diversity. While many publications explored the association between COM values and ROSC or neurological outcomes, few reported outcomes data or investigated COM’s role in influencing treatment. Future research should focus on methodological standardization, broader international representation, and the clinical utility of COM-guided interventions.

## Highlights

• First comprehensive scoping review on cerebral oxygen monitoring in out-of-hospital cardiac arrest.
• Majority of studies used near-infrared spectroscopy (NIRS) and were conducted in emergency departments.
• Prehospital use of cerebral oximetry monitoring remains limited but feasible.
• Few studies evaluated cerebral oximetry monitoring-guided treatment decisions.
• Identified need for standardized methods and broader clinical validation.

## Introduction

Out-of-hospital cardiac arrest (OHCA) is associated with low survival rates, even when return of spontaneous circulation (ROSC) is achieved.^1^ Less than 10 % of patients receiving cardiopulmonary resuscitation (CPR) survive to hospital discharge.^1^ Although all organ systems are affected by cardiac arrest (CA), neurological injury is the leading cause of both late mortality and long-term disability.^2^, ^3^, ^4^, ^5^ Neurological sequelae are common among survivors, and neurological function is considered the key determinant of long-term outcome.^2^, ^4^, ^5^, ^6^ Despite this, cerebral tissue oxygen saturation is not routinely monitored during OHCA.^7^ Tools that allow clinicians to monitor and optimize physiological conditions for ROSC and favorable neurological recovery are crucial to improving OHCA outcomes.^8^ Cerebral tissue oxygen saturation measurements may serve as a supplementary tool to current monitoring methods.^9^

Cerebral oxygen saturation can be measured using both invasive and non-invasive techniques.^10^ Cerebral oxygen monitoring (COM) is a non-invasive technique that measures real-time oxygen saturation in the brain by using near-infrared spectroscopy (NIRS) technology.^8^, ^11^, ^12^ COM detects oxygen levels in brain tissue by leveraging the fact that oxygenated and deoxygenated hemoglobin absorb light differently at specific wavelengths.^8^, ^11^, ^12^ Several cerebral oximeters are currently commercially available, with the earliest devices entering the market in the 1990s.^11^ Most existing COM devices are designed for in-hospital use, such as during surgery, intensive care and neonatal care,^7^, ^10^, ^13^, ^14^ and their use in out-of-hospital settings is sparse.^15^ Although studies have reported COM use during OHCA,^7^, ^13^, ^14^ the extent and nature of its application in these scenarios are not well understood.

The objective of this scoping review (ScR) was to map and describe the types and characteristics of all published studies on the use of COM in patients with OHCA in the prehospital and emergency department (ED) settings. We also aimed to identify knowledge gaps. To meet this objective, research was carried out to answer the primary question:

*What are the types and scope of evidence available that assesses the use of COM in patients with OHCA in prehospital emergency medicine (PHEM) and the ED?*

## Methods

This ScR was conducted in accordance with the JBI Manual for Evidence Synthesis^16^, ^17^, ^18^ and is reported following the Preferred Reporting Items for Systematic reviews and Meta-Analyses ScR (PRISMA-ScR) checklist (Appendix I).^16^, ^19^ An *a priori* protocol was registered and is publicly available in the Open Science Framework (https://doi.org/10.17605/OSF.IO/NRTK5).

### Aims

The primary aim was to assess what evidence exists on the use of COM in OHCA patients in prehospital and ED settings. The secondary aims were to address the following sub-questions:

– What are the characteristics of the populations studied in the published literature?
– What technologies and tools (non-invasive) have been used to monitor cerebral oxygen saturation in patients with OHCA?
– Which outcomes have been evaluated when cerebral oxygen saturation has been monitored during the prehospital or emergency medicine care of patients with OHCA?
– What barriers to the use of COM in patients with OHCA have been reported?
– Has the use of COM influenced the chosen treatment after OHCA and how?

### Search strategy

A comprehensive literature search was performed in MEDLINE, Embase, CINAHL, Cochrane Library and Web of Science from inception through August 8th, 2025, with support from a medical research librarian (SAP). Both thesaurus- and free-text terms were combined to capture studies addressing the two main concepts: ‘cerebral oximetry’ and ‘cardiac/circulatory arrest’. The full search strategies are to be found in the appendices (Appendix II). Document types such as letters and editorials were excluded. Deduplication was conducted in EndNote v21.4 (Clarivate Analytics, PA, USA) (SAP). Forward and backward citation tracking of included studies was conducted using Google Scholar (IB and NG).

### Inclusion and exclusion criteria

Eligibility criteria were defined using the Population, Concept, and Context (PCC) framework. Studies were eligible if they reported on the use of COM (concept) by medical professionals on OHCA patients (population) within prehospital and/or hospital ED settings (context). Studies that reported both OHCA and in-hospital cardiac arrest (IHCA) populations were included only if the OHCA data were reported separately. The review focused exclusively on peer-reviewed primary studies (qualitative, quantitative or mixed methods). No restrictions were applied to language, country or publication date. Records in languages outside the authors’ expertise were translated using the Google Translate document translation tool.

### Source of evidence screening and selection

A pilot screening of 25 records was conducted using the Rayyan web application,^20^ with high inter-reviewer agreement. All records were then screened independently by two sets of reviewers (IB and OU, NG and NKS) at the title/abstract stage and at full-text stage (IB and SEK, NG and NKS). Records were split evenly between the reviewer pairs. Disagreements during title/abstract screening were included for review at the full-text stage. Any discrepancies during full-text review were resolved by a third reviewer (OU). Reasons for exclusion at full-text stage were documented.

### Data extraction

Data were independently extracted in Microsoft Excel v16.92 (WA, USA) by two reviewers (IB and NG) and then consolidated into a shared extraction sheet (Appendix III). Extracted data included study and source characteristics, patient population details, study outcomes, information on COM use, reported barriers, and whether COM influenced treatment decisions. Population characteristics were extracted based on the Utstein OHCA Resuscitation Registry Template recommendations.^21^ All discrepancies or uncertainties were resolved through discussion until consensus was achieved. A guidance form used during the data extraction process is available upon request.

### Analysis and presentation of results

Results are organized according to the review questions and are presented using tables, figures, and narrative descriptions. Common features across studies are reported using descriptive statistics and frequency counts. A basic qualitative analysis was conducted to categorize outcomes and barriers.

Efforts were made to avoid duplicate reporting of the same data across multiple publications,^22^ although this was occasionally difficult to determine. Some separate publications used the same dataset to evaluate different outcomes. In addition, many publications reported on data collected within a similar time frame at the same hospitals, but it remains unclear if the patient populations overlapped. Results and data analysis are therefore based on publication counts, rather than the number of separate studies.

## Results

**Fig. 1:**
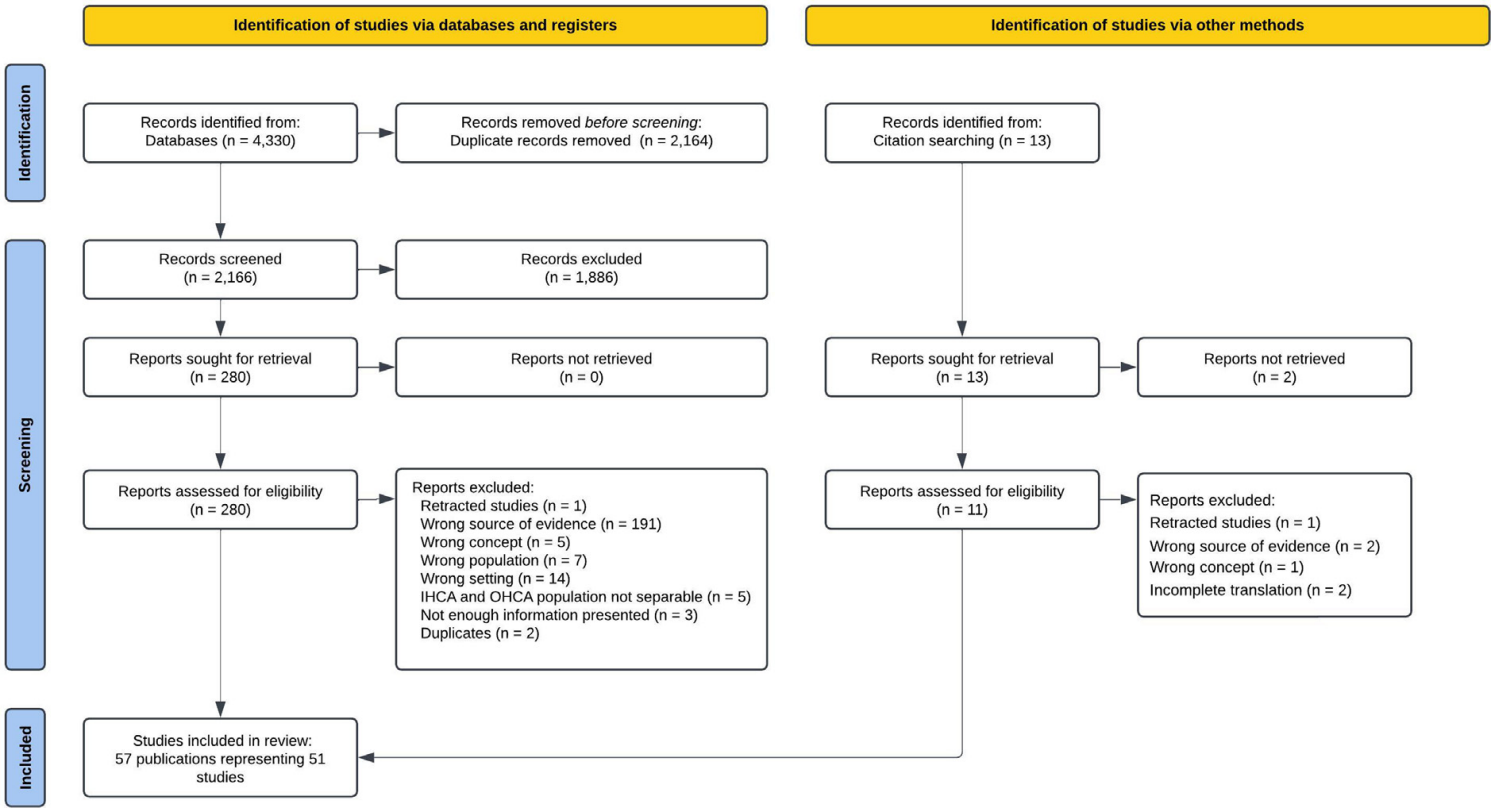
PRISMA flow chart illustrating the inclusion and exclusion process.

The initial database search yielded 4330 records. After removing duplicates, 2166 unique records remained. Following title and abstract screening, 280 records were assessed in full text. Of these, 52 met the inclusion criteria. An additional 13 potentially relevant studies were identified through forward and backward citation screening, of which five were eligible. In total, 57 publications representing 51 studies were included in this ScR (Fig. 1).

References excluded at full-text stage, with reasons, are listed in the appendices (Appendix IV). Google Translate was generally sufficient for assessing eligibility; however, two publications were excluded during data extraction due to incomplete or unclear translation.^23^, ^24^

### Study details and characteristics

Included publications were conducted in Asia (*n* = 32), Europe (*n* = 16) and North America (*n* = 9) (Table 1). Although publication years ranged from 1987 to 2025, all but four were published within the last 15 years. Most publications were cohort designs (prospective or retrospective, *n* = 48), including eight based on multicenter studies: The Japan-Prediction of neurological Outcomes in patients Post cardiac arrest (J-POP) Registry^25^, ^26^, ^27^, ^28^, ^29^, ^30^, ^31^ and the Copernicus Cohort I Study.^9^ Additionally, seven case reports^32^, ^33^, ^34^, ^35^, ^36^, ^37^, ^38^ and two controlled trials were identified (Table 1).^39^, ^40^ The block-randomized and un-blinded study by Berve et al.^39^ compared two types of mechanical CPR devices regarding hemodynamic results, among them cerebral oxygen saturation. Takegawa et al.^40^ published the results from a non-RCT multicenter study, the TripleCPR 16 Study, which assessed the effectiveness of a CPR protocol guided by cerebral oxygen saturation measurements contra the standard rhythm check protocol.

**Table 1:** Table of included studies*.*

Author Year Country	Study design	Aim/Hypothesis	**Setting** Where? Continues after ED?	**Sample size** All patients analyzed Monitored with COM
Abramo et al.^32^ 2014 USA	Case report	Present the potential of cerebral oximetry during arrest and post-arrest phases in pediatric patients.	ED Not reported	2 100 %
Asim et al.^60^ 2014 Turkey	Cohort, prospective	Assess ROSC by evaluating cerebral saturation in OHCA patients.	ED Not reported	23 100 %
Berve et al.^39^ 2022 Norway	Controlled trial	Assess if mechanical active-compression-decompression-CPR would improve hemodynamics and have a similar safety profile to piston-based CPR.	PH Not reported	Intervention: 101 69.3 % Control: 109 60.6 %
Çağlar et al.^61^ 2017 Turkey	Cohort, prospective	Describe cerebral regional oxygen saturation values in pediatric OHCA patients and determine their association with ROSC.	ED No	10 100 %
Drennan et al.^15^ 2019 Canada	Cohort, prospective	Assess the feasibility of the use of NIRS by paramedics during OHCA resuscitation.	PH No	23 83 %
Duvekot et al.^77^ 2015 The Netherlands	Cohort, prospective	Identify OHCA patients at high risk of hyperfibrinolysis, focusing on cerebral oxygenation levels.	ED Not reported	46 100 %
Ehara et al.^71^ 2017 Japan	Cohort, Retrospective	Clarify the relationship between serial changes in cerebral oximetry during ECPR and neurological outcomes.	ED Yes	16 100 %
Fukuda et al.^58^ 2014 Japan	Cohort, prospective	Determine if cerebral oximetry can be used as a static indicator and to identify which values best predict futile resuscitation.	ED Not reported	69 100 %
Genbrugge et al.^9^ 2018 Belgium	Cohort, prospective	Investigate rSO2 values in OHCA patients as a predictor for ROSC, avoiding interference from rSO2 measurements post-ROSC.	PH No	329 100 %
Genbrugge et al.^62^ 2015 Belgium	Cohort, prospective	Measure cerebral oximetry in OHCA patients until ROSC or termination of CPR and investigate differences between those who achieved ROSC and those who did not.	PH No	49 100 %
Hamanaka et al.^45^ 2020 Japan	Cohort, prospective	Assess the feasibility and predictive ability of cerebral oximetry monitoring during CPR by EMT.	PH No	33 100 %
Hayashida et al.^26^ 2014 Japan	Cohort, prospective	Determine whether the estimated cerebral oxy-Hb level is a simple and effective predictor of 90-day neurologic outcomes in patients with PCAS.	ED Not reported	495 (J-POP registry) 100 %
Henningsson et al.^7^ 2022 Sweden	Cohort, prospective	Test the feasibility of using INVOS and Root O3 in addition to using ETCO2 prehospitally and run correlational analyses between rSO2 and ETCO2 to ROSC for both devices.	PH No	27 88.9 %
Heyworth^33^ 1989 England	Case report	Assessment of conjunctival oxygen monitoring as a tool to evaluate cerebral oxygenation during CPR in CA.	ED Not reported	3 (*n* = 2 OHCA, *n* = 1 IHCA) 100 %
Hirose et al.^75^ 2021 Japan	Cohort, retrospective	Observe real-time blood dilution from freshwater drowning as a decrease in HbI values.	ED Not reported	53 100 %
Hirose et al.^50^ 2016 Japan	Cohort, prospective	Clarify prehospital changes in cerebral oximetry in OHCA patients.	PH No	7 100 %
Ito et al.^25^ 2014 Japan	Cohort, prospective	Investigate the association between regional brain oxygen saturation at hospital arrival and 90-day neurological outcomes in OHCA patients.	ED Not reported	672 (J-POP registry) 100 %
Jang et al.^59^ 2023 Korea	Cohort, prospective	Investigate early rSO2 measurements in CPR versus hospital arrival and overall CPR measurements to predict CPR futility in OHCA patients.	ED Not reported	52 100 %
Joo et al.^27^ 2020 Japan	Cohort, retrospective	Investigating the association between rSO2 and neurological outcomes in ECPR patients after OHCA.	ED Not reported	121 (J-POP registry) 100 %
Kalkan et al.^63^ 2015 Turkey	Cohort, prospective	Assess initial and final abdominal and cerebral saturations during CPR using NIRS to determine their correlation with ROSC in OHCA patients.	ED No	34 100 %
Kawaguchi et al.^64^ 2023 Japan	Cohort, retrospective	Evaluate the association between StO2 and ROSC in OHCA patients admitted to the emergency department, and to identify StO2 parameters linked to ROSC.	ED Not reported	108 100 %
Kishihara et al.^55^ 2022 Japan	Cohort, prospective	Evaluate the correlation between MAP and rSO2 during resuscitation as an indicator of chest compression quality.	ED Not reported	37 100 %
Košir et al.^74^ 2023 Slovenia	Cohort, prospective	Test the feasibility of monitoring skeletal muscle rSO2 during CPR after OHCA and explore its relationship with brain rSO2.	PH No	20 95.0 %
Koyama et al.^34^ 2018 Japan	Case report	Report a case where NIRO-pulse evaluated CPR efficacy, maintaining TOI, and leading to successful social activity.	ED Not reported	1 100 %
Koyama et al.^56^ 2013 Japan	Cohort, prospective	Evaluate CPR quality using ΔcHb and TOI readings and their correlation with ROSC.	ED Not reported	15 100 %
Levis et al.^76^ 2022 Japan	Cohort, prospective	Investigating the feasibility of ultrasound-facilitated REBOA-catheter placement during CPR in an emergency department.	ED Not reported	15 60 %
Matsuyama et al.^35^ 2022 Japan	Case report	Describe cases of NIRO-Pulse for physiological monitoring during CPR.	ED Not reported	4 100 %
Meex et al.^41^ 2013 Belgium	Cohort, prospective	Test the feasibility of FORE-SIGHT and EQUANOX in the CPR setting.	PH No	14 (*n* = 9 OHCA, *n* = 5 IHCA) 100 %
Müllner et al.^72^ 1995 Austria	Cohort, prospective	Investigate if rSO2 can predict outcomes and reflect cerebral oxygen metabolism during and after CPR, and its relation to blood pressure and pulse oximetry.	ED Not reported	18 100 %
Nakatani et al.^28^, ^29^ 2018 Japan	Cohort, retrospective	Examine the effectiveness of TTM at 32–34 °C, considering cerebral injury and circulation via rSO2 on hospital arrival.	ED Not reported	431 (J-POP registry) 100 %
Nelskylä et al.^73^ 2022 Finland	Cohort, prospective	Assess hyperoxia during CPR by HEMS and study the correlation of intra-arrest NIRS values with perfusion pressure, ETCO2, blood pressure, paO2, and paCO2.	PH No	97 97.3 %
Newman et al.^48^ 2004 USA	Cohort, prospective	Determine the feasibility of field COx readings in OHCA patients and the impact of ventilatory rate on cerebral perfusion.	PH Not reported	16 100 %
Nishiyama et al.^30^ 2015a Japan	Cohort, prospective	Compare rSO2 values in patients during and after resuscitation upon hospital arrival to estimate CPR quality and neurological prognostication.	ED Not reported	1921 (J-POP registry) 100 %
Nishiyama et al.^31^ 2015b Japan	Cohort, prospective	Evaluate rSO2 monitoring for estimating brain damage severity and prognoses in OHCA patients.	ED Not reported	1195 (J-POP registry) 100 %
Ogawa et al.^53^ 2015 Japan	Cohort, prospective	Evaluate changes in rSO2 during CPR and the effects of LDB-CPR on rSO2.	ED Not reported	34 100 %
Onishi et al.^36^ 2023 Japan	Case report	To present four cases of monitoring arterial and venous pressure and SctO2 during CPR in adult OHCA patients.	ED Not reported	4 100 %
Prosen et al.^65^ 2018 Slovenia	Cohort, prospective	Evaluate and compare continuous rSO2 measurements during OHCA, particularly around the time of ROSC.	PH and ED Yes	53 100 %
Rola et al.^37^ 2021 Canada	Case report	Case report of a 36-year-old woman with OHCA. REBOA catheter use led to a ROSC and improved arterial blood pressure, end-tidal CO2, and cerebral oximetry.	ED Not reported	1 100 %
Rutherford et al.^38^ 1987 USA	Case report	Case report highlights the value of PcjO2 and PcjO2/CaO2 ratio as indicators of compromised cerebral oxygenation and perfusion after CPR.	ED Not reported	1 100 %
Sakaguchi et al.^66^ 2022 Japan	Cohort, retrospective	Investigate SnO2 values during CPR to test if they predict ROSC in patients arriving at the ED.	ED Not reported	42 100 %
Sakai et al.^78^ 2021 Japan	Cohort, prospective	Evaluate the association between serial rSO2 changes in the prehospital setting and patient outcomes.	PH Not reported	87 100 %
Schewe et al.^46^ 2014 Germany	Cohort, prospective	Investigating the feasibility of NIRS monitoring during OHCA in a physician-staffed emergency medical service.	PH No	10 100 %
Shin et al.^67^ 2022 USA	Cohort, prospective	Assessing cerebral oximetry during initial first responder resuscitation to determine its association with ROSC and functional survival following OHCA.	PH Not reported	59 100 %
Shin et al.^52^ 2025 USA	Cohort, prospective	Assess regional cerebral oxygen saturation (rSO_2_) during resuscitation to explore its association with ROSC and functional survival.	PH Not reported	93 100 %
Singer et al.^47^ 2015 USA	Cohort, retrospective	Describe the feasibility of real-time cerebral oximetry in ED patients with out-of-hospital SCA and its association with rSO2, ROSC, and survival.	ED Not reported	59 100 %
Singer et al.^70^ 2018 USA	Cohort, prospective	Compare the accuracy of ETCO2 and rSO2 in predicting ROSC in ED patients with OHCA.	ED Not reported	100 100 %
Storm et al.^51^ 2016 Germany	Cohort, prospective	Understand brain oxygen saturation during CPR by measuring frontal brain rSO2 in OHCA patients using NIRS.	PH and ED Yes	29 100 %
Tajima et al.^49^ 2015 Japan	Cohort, prospective	Create a system to monitor rSO2 in prehospital cardiac arrest patients and clarify rSO2 changes during CPR.	PH Not reported	9 100 %
Takegawa et al.^42^ 2019 Japan	Cohort, retrospective	Evaluate the relationship between rSO2 increase during CPR on hospital arrival and the occurrence of ROSC to find new prediction criteria.	ED Not reported	90 100 %
Takegawa et al.^40^ 2021 Japan	Controlled trial	Evaluate if a CPR algorithm with rSO2 monitoring, but without a rhythm check every 2 min, improves ROSC rates in OHCA patients.	ED Not reported	Intervention: 225 100 % Control: 86 (Historical control cohort from Takegawa et al.^42^) 100 %
Taniguchi et al.^68^ 2022 Japan	Cohort, prospective	Evaluate if temporal changes in peripheral rSO2 are more sensitive predictors of ROSC in OHCA patients than cerebral rSO2, leading to more effective monitoring.	ED Not reported	145 100 %
Tsukuda et al.^69^ 2019 Japan	Cohort, prospective	Evaluate if adding initial TOI to Utstein core variables could predict ROSC or non-ROSC, and to identify 'ECPR zone' and 'TOR zone' using TOI cut-off values.	ED Not reported	117 100 %
Tsukuda et al.^57^ 2021 Japan	Cohort, prospective	Examine the association between ΔStO2 and ROSC, and between ΔStO2 and CPR quality.	PH No	81 100 %
Yagi et al.^43^ 2020 Japan	Cohort, prospective	Evaluate if the NIRO-CCR1 is as clinically useful as the NIRO-200NX.	ED Not reported	20 (*n* = 19 from Yagi et al.^44^) 100 %
Yagi et al.^44^ 2016 Japan	Cohort, prospective	Evaluate if NIRS allows the detection of ROSC during chest compression without interruption.	ED Not reported	19 100 %
Yagi et al.y^54^ 2013 Japan	Cohort, prospective	Evaluate changes of cerebral blood oxygenation during ECPR using NIRS.	ED Not reported	15 100 %

**Abbreviations**.

CA (cardiac arrest), CaO2 (arterial oxygen content), COx (cerebral oximetry), CPR (cardiopulmonary resuscitation), ECPR (extracorporeal CPR), EMT (emergency medical technician), ETCO2 (end-tidal carbon dioxide), HEMS (Helicopter Emergency Medical Services), HbI (hemoglobin index), LDB-CPR (Load Distributing Band-CPR), MAP (mean arterial pressure), NIRS (near infrared spectroscopy), OHCA (out-of-hospital cardiac arrest), oxy-Hb (oxyhemoglobin), paO2 (partial pressure of oxygen), paCO2 (partial pressure of carbon dioxide), PCAS (post cardiac arrest syndrome), PcjO2 (conjunctival oxygenation), REBOA (resuscitative endovascular balloon occlusion of the aorta), ROSC (return of spontaneous circulation), rSO2 (regional cerebral oxygen saturation), SctO2 (cerebral tissue oxygen saturation), SnO2 (cerebral tissue oxygen saturation), StO2 (cerebral oxygen saturation), TOI (tissue oxygenation index), TOR (termination of resuscitation), TTM (targeted temperature management), ΔcHb (total hemoglobin concentration change).

Apart from the three multicenter studies, small sample sizes were common. About 45 % of the publications had a sample size of 30 or less. Two publications included both OHCA and IHCA patients. 33, 41 Most publications were conducted in the ED (*n* = 38), while 17 publications involved prehospital initiation of COM (Table 1). Two publications spanned both settings, and three reported continuation of COM beyond the ED (Table 1). Full study details are provided in Table 1; the complete data extraction is available in the appendices (Appendix III).

Several publications from Japan reported overlapping populations. Seven were based on J-POP data,^25^, ^26^, ^27^, ^28^, ^29^, ^30^, ^31^ including the complimentary data article by Nakatani et al.^28^, ^29^ Two publications by Takegawa et al.^40^, ^42^ and two by Yagi et al.^43^, ^44^ were also based on overlapping cohorts.

### Study aims and objectives

While study aims varied, several recurring objectives were identified. There were publications whose aims spanned two or more of the identified common features. Seven publications focused on assessing the feasibility of COM, either in a prehospital or ED setting, using various devices.^7^, ^15^, ^41^, ^45^, ^46^, ^47^, ^48^ Ten publications monitored changes in cerebral oxygenation during CPR,^30^, ^33^, ^35^ mechanical CPR^53^ or extracorporeal CPR.^54^ Other aims included evaluating CPR quality (*n* = 4),^30^, ^55^, ^56^, ^57^ CPR efficacy (*n* = 1)^34^ or CPR futility (*n* = 2).^58^, ^59^ Approximately one-third (*n* = 20) of the publications examined associations between COM values and outcomes such as ROSC^9^, ^42^, ^47^, ^52^, ^57^, ^60^, ^61^, ^62^, ^63^, ^64^, ^65^, ^66^, ^67^, ^68^, ^69^, ^70^ or neurological recovery,^25^, ^26^, ^30^, ^31^, ^52^, ^71^ exploring the potential of COM as a prognostic tool in CA patients. Several studies compared COM values with physiological parameters like hemodynamics,^55^, ^72^, ^73^ end-tidal CO_2,_^7^, ^70^, ^73^ or tissue oxygenation in other body parts.^63^, ^68^, ^74^ The goal of the former studies, beyond comparing the parameters, included investigating how COM and other physiological parameters correlate during resuscitation, and comparing the quality of COM versus the other parameters in predicting ROSC. Three publications did not present explicit COM-related aims.^39^, ^75^, ^76^ Table 1 presents the aims of all included publications in brief form.

### Populations characteristics

Most publications included adult OHCA patients, with 61 % (*n* = 35) enrolling only individuals aged ≥18 years.^7^, ^15^, ^25^, ^26^, ^27^, ^29^, ^30^, ^31^, ^36^, ^39^, ^45^, ^46^, ^47^, ^52^, ^54^, ^55^, ^56^, ^57^, ^58^, ^59^, ^62^, ^63^, ^64^, ^65^, ^66^, ^67^, ^68^, ^69^, ^70^, ^73^, ^74^, ^76^, ^77^, ^78^ Only two publications focused specifically on pediatric patients.^32^, ^61^ Males were overrepresented across publications; just eight publications (14.3 %) included ≥50 % female participants, often in subgroups only.^32^, ^37^, ^49^, ^60^, ^61^, ^63^, ^75^, ^78^ Patient cohorts were frequently stratified by outcome (e.g., ROSC versus non-ROSC) or intervention type (*n* = 14).^7^, ^9^, ^39^, ^40^, ^42^, ^51^, ^52^, ^57^, ^59^, ^65^, ^68^, ^71^, ^75^, ^78^ CA etiology was variably reported, with most publications including all causes, while trauma was a common exclusion criterion (*n* = 31).^7^, ^9^, ^25^, ^26^, ^27^, ^29^, ^30^, ^31^, ^39^, ^40^, ^45^, ^46^, ^48^, ^51^, ^52^, ^53^, ^55^, ^56^, ^57^, ^58^, ^59^, ^61^, ^62^, ^63^, ^65^, ^68^, ^69^, ^73^, ^74^, ^76^, ^77^ Timing of COM ranged from arrest onset to the post-resuscitation phase, although many studies excluded patients who had already achieved ROSC (*n* = 21).^9^, ^40^, ^42^, ^43^, ^44^, ^47^, ^48^, ^49^, ^58^, ^59^, ^62^, ^64^, ^66^, ^71^, ^73^, ^74^, ^75^, ^77^, ^78^ Mechanical CPR was reported in 12 publications,^7^, ^9^, ^36^, ^37^, ^39^, ^40^, ^42^, ^46^, ^47^, ^53^, ^73^, ^76^ but consistently used in all included patients in only nine publications.^7^, ^36^, ^37^, ^39^, ^40^, ^42^, ^47^, ^53^, ^76^ Post-CA interventions were described in 19 publications,^9^, ^25^, ^26^, ^27^, ^29^, ^30^, ^31^, ^32^, ^34^, ^37^, ^43^, ^44^, ^46^, ^50^, ^51^, ^54^, ^69^, ^71^, ^77^ and 21 publications explicitly followed Utstein-style reporting guidelines.^9^, ^25^, ^26^, ^27^, ^29^, ^30^, ^31^, ^44^, ^45^, ^47^, ^52^, ^54^, ^57^, ^58^, ^59^, ^62^, ^65^, ^67^, ^69^, ^70^, ^78^

### Technologies and tools

**Fig. 2:**
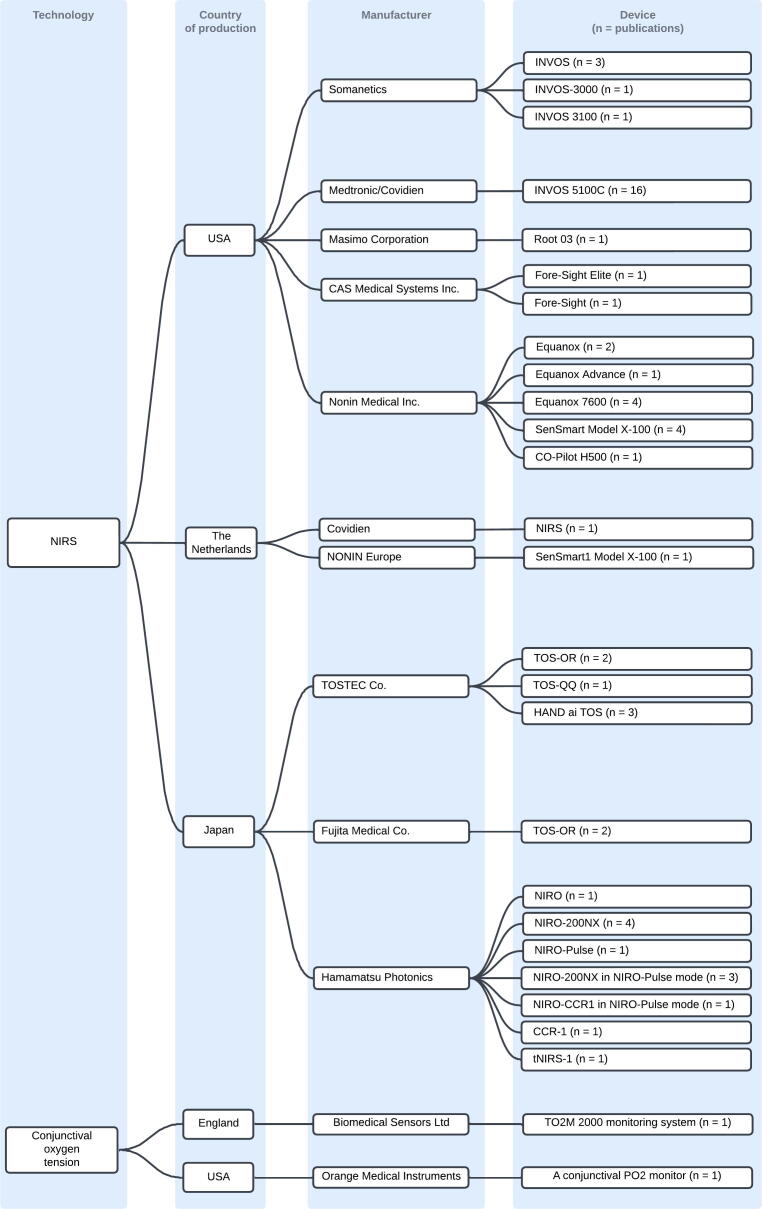
Overview of technologies, countries of production, manufacturers, devices and the number of publications reporting each device.

Two technologies were used to measure cerebral oxygenation: NIRS and conjunctival oxygen tension. NIRS was by far the most common (*n* = 55), including one publication using a time-domain NIRS variant.^64^ Two older publications used conjunctival oxygen sensors.^33^, ^38^ Twenty-seven different COM devices were identified. Fig. 2 illustrates the types of technology, country of manufacture, manufacturers, and the number of publications using each device.

## Outcomes evaluated

**Fig. 3:**
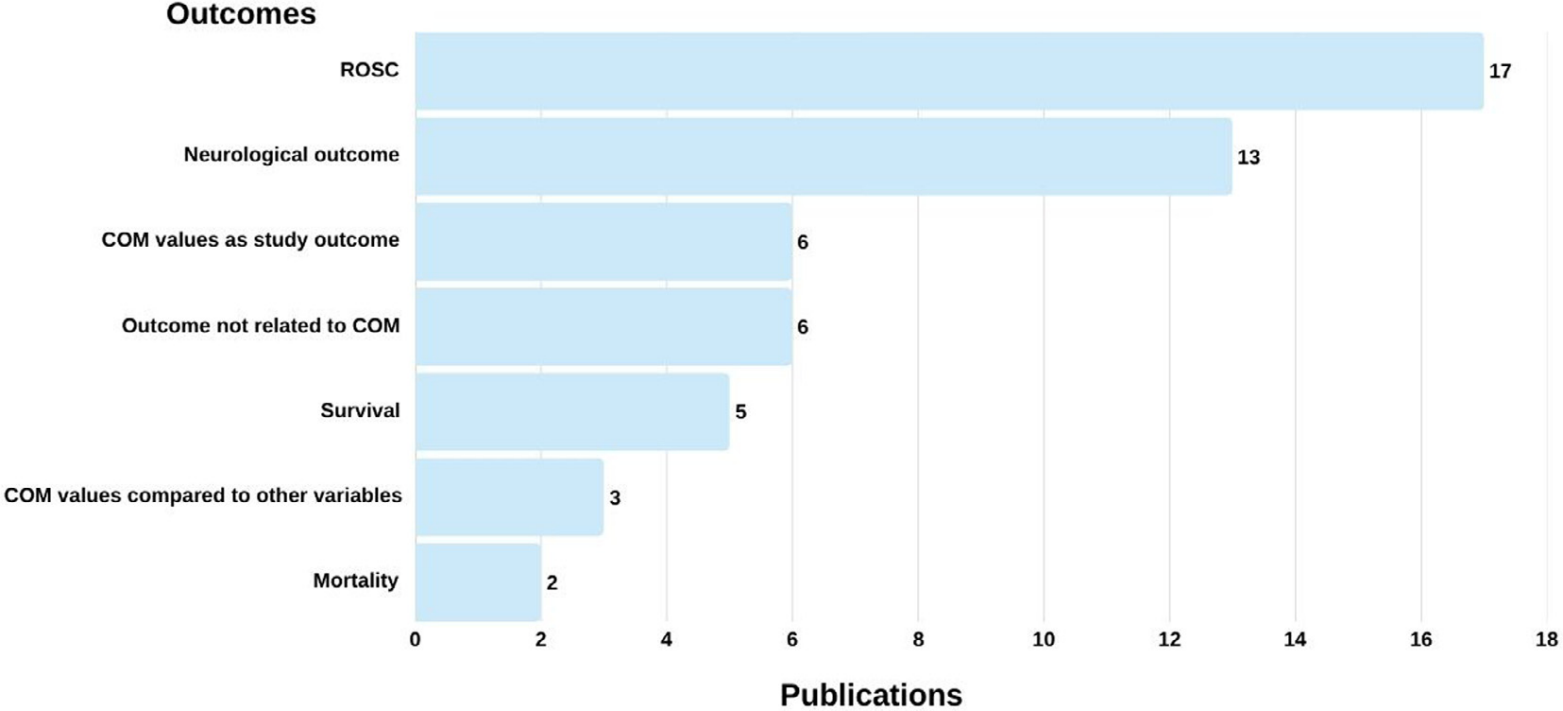
Summary of outcomes reported.

Half of the included publications (*n* = 29) reported one or more defined outcomes,^9^, ^25^, ^26^, ^27^, ^29^, ^30^, ^31^, ^39^, ^40^, ^45^, ^47^, ^51^, ^52^, ^55^, ^57^, ^58^, ^59^, ^62^, ^65^, ^66^, ^67^, ^69^, ^70^, ^72^, ^73^, ^74^, ^75^, ^76^, ^78^ which could be grouped into seven categories (Fig. 3). Sixteen publications assessed more than one outcome. The most frequently evaluated outcome was ROSC (*n* = 17),^9^, ^39^, ^40^, ^45^, ^47^, ^52^, ^57^, ^58^, ^59^, ^62^, ^65^, ^66^, ^67^, ^69^, ^70^, ^74^, ^78^ followed by neurological outcome (*n* = 13).^9^, ^25^, ^26^, ^27^, ^29^, ^30^, ^31^, ^51^, ^52^, ^65^, ^67^, ^72^, ^78^ Follow-up time points for neurological outcomes varied (e.g., 90 days post hospital admission,^26^ ICU discharge,^51^, ^52^ 180 days post CA.^9^ Other evaluated outcomes included COM values as primary outcome (*n* = 6), 39, 65, 66, 73, 76, 78 survival (*n* = 5),^39^, ^45^, ^47^, ^65^, ^78^ and mortality (*n* = 2).^29^, ^72^ Three publications compared COM readings with physiological markers such as mean arterial blood pressure (MAP) and systolic arterial blood pressure (SAP),^55^ regional oxygen saturation (rSO_2_) in skeletal muscle,^74^ and chest compression rate.^57^

### Reported barriers to COM use

Of the 57 included publications, 28 % reported barriers to the use of COM, specifically in relation to NIRS devices. These barriers were broadly categorized into four groups: technical, application-related, physical, and personnel-related factors (Table 2). Technical barriers were the most commonly reported barrier and appeared in twelve publications,^7^, ^9^, ^25^, ^41^, ^43^, ^48^, ^52^, ^57^, ^61^, ^65^, ^69^, ^74^ involving issues such as power supply, monitor delays, missing or unreadable values, and connectivity problems between sensors and monitors. Difficulties with sensor application were reported in eight publications.^7^, ^9^, ^48^, ^57^, ^64^, ^66^, ^69^, ^72^ These included sensor displacement, attachment failure, or facial trauma impeding placement. Physical constraints were also described in eight publications,^7^, ^25^, ^30^, ^31^, ^41^, ^43^, ^48^, ^65^ relating to device size and weight, lack of portability, limited space during resuscitation, and shortages of disposable probes. Barriers related to staffing were reported in five publications,^7^, ^25^, ^48^, ^64^, ^65^, such as insufficient personnel and concerns that monitoring diverted attention from advanced life support efforts.

**Table 2:** Barriers identified in the included literature.

Technical barriers (*n* = 12)	Application barriers (*n* = 8)	Physical barriers (*n* = 8)	Staff barriers (*n* = 5)
Low battery capacity (*n* = 2) Lack of power source (*n* = 1) Monitor charging (*n* = 1) No registered values (*n* = 2) No value displayed (*n* = 1) Monitor start-up delay (*n* = 2) Low internal storage time (*n* = 1) Connection problem sensor/monitor (*n* = 2) Equipment malfunction (*n* = 1) Technical problems with COM (*n* = 2)	Sensor attachment failure (*n* = 3) Sensor fell off (*n* = 1) Frontal head trauma (*n* = 1) Lack of forehead access (*n* = 1) Deviation in probe attachment introduce measurement error (*n* = 1) Sensor displacement (*n* = 1)	Heavy device (*n* = 3) Big device (*n* = 1) No portable monitor (*n* = 3) Difficult to carry (*n* = 1) Limited physical space (*n* = 1) Patient transport (*n* = 1) Shortage of disposable probes (*n* = 1)	Insufficient personnel (*n* = 4) The monitoring shifted focus away from ALS (*n* = 1)

**Abbreviations**.

ALS (advanced life support), COM (cerebral oximetry monitoring).

### Influence of COM on the treatment

In four publications, COM readings were explicitly reported to influence clinical decision-making.^32^, ^34^, ^40^, ^47^ In two case reports, favorable cerebral oxygenation values were used to justify continued resuscitation despite other negative prognostic indicators.^32^, ^34^ In the publication by Takegawa et al.,^40^ a CPR protocol based on rSO_2_ trends, rather than traditional rhythm checks, showed improved ROSC rates. In a publication by Singer et al.,^47^ patients were selected for mechanical CPR based on COM thresholds. Mechanical CPR was performed when cerebral oxygen saturation on arrival fell below a predefined threshold, which applied to all the patients included in this publication.^47^ Consequently, the publication was observational rather than interventional in nature.

Seven additional publications suggested that COM may have unintentionally influenced clinical decisions, particularly regarding continuation or cessation of resuscitation, because clinicians were not blinded to the readings.^9^, ^25^, ^30^, ^31^, ^50^, ^58^, ^78^ Only six publications stated explicitly that the treating teams were blinded to COM values.^45^, ^46^, ^52^, ^59^, ^65^, ^67^

## Discussion

This ScR identified a considerable body of literature on COM in OHCA patients within prehospital and ED settings, with the majority of publications published in the last 15 years. The number of included publications exceeded expectations and was markedly dominated by research from Japan, accounting for more than half of the publications. Most publications were small-sample observational designs focused on changes in cerebral oxygenation during CA and resuscitation, as well as associations between COM values and various patient outcomes.

### Country of origin

Japan is the primary contributor to COM research in OHCA. In Japan, emergency medical technicians are generally not permitted to terminate CPR in the field, resulting in more frequent transport and ongoing resuscitation of OHCA patients compared to practices in Europe and North America.^45^, ^58^, ^59^ This resource-intensive approach may explain the regional concentration of publications and underlines the need for improved prehospital prognostic tools. The CPR guidelines in South Korea are equivalent to the guidelines in Japan.^59^ A paper from South Korea, by Jang et al., emphasized the importance of more precise guidelines to help identify patients in whom resuscitative efforts should be intensified versus those where termination may be appropriate.^59^ While such tools are relevant globally, they are particularly pressing in settings where all OHCA patients are brought to the ED. As highlighted by Nakatani et al., an overrepresentation of patients with poor prognosis in Japanese studies may risk underestimating the clinical value of COM,^31^ raising questions about the generalizability of such findings to regions with different resuscitation protocols.

### Setting for monitoring

The majority of publications were conducted in the ED rather than the prehospital setting. However, ED-based COM typically begins well after the onset of arrest, excluding patients with short resuscitation times and early ROSC—those with potentially favorable outcomes. ^79^ Including these patients would provide valuable data from a broader spectrum of clinical trajectories.^62^ In 2018, Burell et al. called for further research into the feasibility of COM use by paramedics.^80^ Since then, several papers have confirmed the feasibility of prehospital COM application.^15^, ^45^ Notably, the J-POP investigators initially intended to include prehospital COM, but the lack of portable devices prevented its implementation.^25^ Such devices have since become available, opening the door for broader use.^49^

### Population characteristics

The included publications reported highly variable population characteristics. Inconsistencies in data reporting made synthesis challenging. Adopting standardized frameworks such as the Utstein-style guidelines could improve consistency in future research: only 20 of the included publications reported using these standards. Two publications addressed pediatric OHCA, mirroring findings from a ScR by Kool et al., which also identified only two COM-related pediatric studies.^81^ While additional pediatric COM studies exist, they primarily focus on ICU monitoring^82^ or combine in-hospital and out-of-hospital data that are not distinguishable.^83^, ^84^ Given the more frequent use of COM in pediatric intensive care and surgery,^85^, ^86^ its role in pediatric OHCA deserves further exploration. The sparse number of studies on the pediatric OHCA population may be due to the older population having a higher prevalence of OHCA and for this reason; COM in pediatric OHCA has not been a prioritized research group. Similarly, the predominance of males across publications likely reflects the higher incidence of OHCA among men.

### COM technologies and devices

The predominance of NIRS across publications is unsurprising given its many advantages. NIRS offers non-invasive, real-time, continuous monitoring that is independent of pulsatile flow—key attributes of an ideal monitoring device as described by Meex et al.^41^ Despite these advantages, the diversity of devices used across studies presents challenges for interpretation. Although most devices were NIRS-based, they varied significantly in design, including differences in wavelength numbers, photodetector configuration, internal algorithms, and calibration methods.^8^, ^41^ These differences complicate cross-study comparisons and should be considered when synthesizing data or developing consensus on COM’s clinical utility.^8^ Publications also varied in how and when COM values were measured—before, during, or after ROSC—and in whether they focused on static values, dynamic trends, or both. Several authors raised questions about which type of values are most useful and whether different types of values serve different clinical purposes, such as predicting ROSC, assessing CPR quality, or forecasting neurological outcomes.^7^, ^9^, ^51^ While some SRs have explored the associations between initial or mean rSO_2_ values and outcomes, 13, 14, 87 a comprehensive synthesis of the role of dynamic measurements is still lacking. To support comparability and clinical translation, future studies should aim to standardize measurement protocols, timing, and outcome reporting across different COM devices.

### Barriers to using COM in OHCA

Barriers to COM use were reported in a relatively small number of publications. Much of the existing literature emerges from experienced research groups familiar with the technology. For example, Newman et al.,^48^ among the first to publish on COM in OHCA, reported limited experience and highlighted multiple technical challenges. In contrast, Singer et al. noted that their regular COM use in the ED may limit generalizability.^70^ Some publications had additional personnel assigned to manage COM, or may have been subject to sampling bias, potentially underreporting real-world implementation challenges. Further research is needed under typical clinical conditions to assess the feasibility and limitations of COM outside expert settings.

### Treatment pathway

Few publications examined the role of COM in guiding clinical decision-making. This likely reflects the early stage of implementation, with most research to date focusing on feasibility. Moreover, there remains no consensus on which clinical endpoints to use in such studies. A significant number of included publications did not report outcome measures, possibly due to incomplete or selective reporting common in observational designs.^88^ This creates risks of bias and may hinder causal interpretation.^88^ To fully evaluate COM’s impact on clinical decision-making and patient outcomes, larger comparative studies are needed.

### Limitations

Despite a comprehensive literature search, some relevant publications were identified only through citation tracking, suggesting that additional publications may have been missed. The review excluded grey literature and ongoing studies, which may have limited the scope of included evidence. Moreover, some double counting of data may have occurred, as several publications appeared to use overlapping datasets. While efforts were made to identify and avoid duplication, it is not possible to confirm that all such cases were resolved. Finally, the initial database search identified numerous abstracts from meetings and conferences. Many of these abstracts presented interesting findings and did not have a corresponding published article to date. It indicates a promising future for this research field if these studies are to be published in peer reviewed journals.

## Conclusion

The current literature on COM in OHCA is dominated by small observational studies from Japan, with most research conducted in the ED rather than prehospital settings. The technology most used is NIRS, and publications generally involved older male patients with non-traumatic causes of arrest. While many publications explored the association between COM values and ROSC or neurological outcomes, few reported outcomes data or investigated COM’s role in influencing treatment. Barriers to COM use were infrequently reported, and its clinical application remains limited. Future research should focus on standardization of methodology, broader geographic representation, and the potential clinical utility of COM-guided interventions.

### Statement

During the preparation of this work the author(s) used Open AI ChatGPT version 4.0 in order to improve readability and language. After using this tool/service, the author(s) reviewed and edited the content as needed and take(s) full responsibility for the content of the published article.

## CRediT authorship contribution statement

**Ingjerd Baugstø:** Writing – review & editing, Writing – original draft, Visualization, Software, Methodology, Investigation, Formal analysis, Data curation, Conceptualization. **Nora Gjesdal:** Writing – review & editing, Writing – original draft, Visualization, Software, Methodology, Investigation, Formal analysis, Data curation, Conceptualization. **Sarah Elizabeth King:** Writing – review & editing, Writing – original draft, Validation, Supervision, Methodology, Investigation, Formal analysis, Data curation, Conceptualization. **Sindre Andre Pedersen:** Writing – review & editing, Methodology, Investigation, Data curation. **Lars Petter Bache-Wiig Bjørnsen:** Writing – review & editing, Conceptualization. **Nils Kristian Skjærvold:** Writing – review & editing, Supervision, Investigation, Data curation, Conceptualization. **Oddvar Uleberg:** Writing – review & editing, Writing – original draft, Supervision, Project administration, Investigation, Data curation, Conceptualization.

## Funding

This research did not receive any specific grant from funding agencies in the public, commercial, or not-for-profit sectors.

## Declaration of competing interest

The authors declare the following financial interests/personal relationships which may be considered as potential competing interests: Authors IB, NG, SEK, SAP, and LPBWB state no conflict of interest. Authors NKS and OU state that they has a stock ownership in Cate Medical AS. Cate Medical AS was established in June 2023 by NKS (founder) and OU (co-founder), to further develop and market the OxyCate sensor system / concept. The share capital totals approximately 2600 Euros. There are no employees or investors so far. No commercial product is available so far.
