# Supplementary material for: Cerebral oxygen monitoring during out-of-hospital cardiac arrest: A scoping review
Source: Resusc Plus. 2025 Sep 3;26:101082. doi: 10.1016/j.resplu.2025.101082 (PMC12744649; doi:10.1016/j.resplu.2025.101082)
Supplement: Supplementary Data 2 [file mmc2.pdf]

Appendix II: Documentation of search strategies for the bibliographic databases

MEDLINE via EBSCOhoste

#	Query
S1	(MH "Brain+") AND (MH "Oximetry+")
S2	TI (((cerebr* OR brain* OR crani*) N1 (oxigen* OR oxygen* OR oximet* OR oxymet*))) OR AB (((cerebr* OR brain* OR crani*) N1 (oxigen* OR oxygen* OR oximet* OR oxymet*))) OR CI (((cerebr* OR brain* OR crani*) N1 (oxigen* OR oxygen* OR oximet* OR oxymet*)))
S3	TI (((cerebr* OR brain* OR crani*) N2 'near infrared')) OR AB (((cerebr* OR brain* OR crani*) N2 'near infrared')) OR CI (((cerebr* OR brain* OR crani*) N2 'near infrared'))
S4	S1 OR S2 OR S3
S5	(MH "Heart Arrest+")
S6	(MH "Return of Spontaneous Circulation")
S7	(MH "Cardiopulmonary Resuscitation+")
S8	TI (asystole) OR AB (asystole) OR CI (asystole)
S9	TI (asystolia) OR AB (asystolia) OR CI (asystolia)
S10	TI (asystoly) OR AB (asystoly) OR CI (asystoly)
S11	TI ("cardiac arrest") OR AB ("cardiac arrest") OR CI ("cardiac arrest")
S12	TI ("circulation arrest") OR AB ("circulation arrest") OR CI ("circulation arrest")
S13	TI ("circulatory arrest") OR AB ("circulatory arrest") OR CI ("circulatory arrest")
S14	TI ("heart asystole") OR AB ("heart asystole") OR CI ("heart asystole")
S15	TI ("heart standstill") OR AB ("heart standstill") OR CI ("heart standstill")
S16	TI ("heart arrest") OR AB ("heart arrest") OR CI ("heart arrest")
S17	TI ("recovery of spontaneous circulation") OR AB ("recovery of spontaneous circulation") OR CI ("recovery of spontaneous circulation")
S18	TI ("restoration of spontaneous circulation") OR AB ("restoration of spontaneous circulation") OR CI ("restoration of spontaneous circulation")
S19	TI (ROSC) OR AB (ROSC) OR CI (ROSC)
S20	TI ("return of spontaneous circulation") OR AB ("return of spontaneous circulation") OR CI ("return of spontaneous circulation")
S21	TI ("spontaneous circulation return") OR AB ("spontaneous circulation return") OR CI ("spontaneous circulation return")
S22	TI (CPR) OR AB (CPR) OR CI (CPR)

S23	TI ("cardio pulmonary resuscitation") OR AB ("cardio pulmonary resuscitation") OR CI ("cardio pulmonary resuscitation")
S24	TI ("cardiopulmonary resuscitation") OR AB ("cardiopulmonary resuscitation") OR CI ("cardiopulmonary resuscitation")
S25	TI ("chest compression") OR AB ("chest compression") OR CI ("chest compression")
S26	TI (reanimation) OR AB (reanimation) OR CI (reanimation)
S27	TI (resuscitation) OR AB (resuscitation) OR CI (resuscitation)
S28	S5 OR S6 OR S7 OR S8 OR S9 OR S10 OR S11 OR S12 OR S13 OR S14 OR S15 OR S16 OR S17 OR S18 OR S19 OR S20 OR S21 OR S22 OR S23 OR S24 OR S25 OR S26 OR S27
S29	S4 AND S28

Embase via Embase.com

#	Query
#1	'cerebral oximeter'/exp
#2	'cerebral oximetry'/exp
#3	'cerebral oximetry index'/exp
#4	'brain'/exp AND ('oximetry'/exp OR 'oximeter'/exp)
#5	((cerebr* OR brain* OR crani*) NEAR/2 (oxigen* OR oxygen* OR oximet* OR oxymet*)):ti,ab,kw
#6	((cerebr* OR brain* OR crani*) NEAR/3 'near infrared'):ti,ab,kw
#7	#1 OR #2 OR #3 OR #4 OR #5 OR #6
#8	'heart arrest'/exp
#9	'return of spontaneous circulation'/exp
#10	'resuscitation'/exp
#11	'asystole':ti,ab,kw
#12	'asystolia':ti,ab,kw
#13	'asystoly':ti,ab,kw
#14	'cardiac arrest':ti,ab,kw
#15	'circulation arrest':ti,ab,kw
#16	'circulatory arrest':ti,ab,kw
#17	'heart asystole':ti,ab,kw
#18	'heart standstill':ti,ab,kw
#19	'heart arrest':ti,ab,kw
#20	'recovery of spontaneous circulation':ti,ab,kw
#21	'restoration of spontaneous circulation':ti,ab,kw
#22	'rosc':ti,ab,kw
#23	'return of spontaneous circulation':ti,ab,kw
#24	'spontaneous circulation return':ti,ab,kw
#25	'cpr':ti,ab,kw
#26	'cardio pulmonary resuscitation':ti,ab,kw
#27	'cardiopulmonary resuscitation':ti,ab,kw
#28	'chest compression':ti,ab,kw
#29	'reanimation':ti,ab,kw

#30	'resuscitation':ti,ab,kw
#31	#8 OR #9 OR #10 OR #11 OR #12 OR #13 OR #14 OR #15 OR #16 OR #17 OR #18 OR #19 OR #20 OR #21 OR #22 OR #23 OR #24 OR #25 OR #26 OR #27 OR #28 OR #29 OR #30
#32	#7 AND #31
#32	editorial:it OR letter:it
#32	#32 NOT #33

Cochrane Library

ID Search Hits

#1 MeSH descriptor: [Brain] explode all trees

#2 MeSH descriptor: [Oximetry] explode all trees

#3 #1 AND #2

#4 ((cerebr* OR brain* OR crani*) NEAR/1 (oxigen* OR oxygen* OR oximet* OR oxymet*)):ti,ab,kw (Word variations have been searched)

#5 ((cerebr* OR brain* OR crani*) NEAR/2 "near infrared"):ti,ab,kw (Word variations have been searched)

#6 #3 OR #4 OR #5

#7 MeSH descriptor: [Heart Arrest] explode all trees

#8 MeSH descriptor: [Return of Spontaneous Circulation] explode all trees

#9 MeSH descriptor: [Cardiopulmonary Resuscitation] explode all trees

#10 (asystole):ti,ab,kw (Word variations have been searched)

#11 (asystolia):ti,ab,kw (Word variations have been searched)

#12 (asystoly):ti,ab,kw (Word variations have been searched)

#13 ("cardiac arrest"):ti,ab,kw (Word variations have been searched)

#14 ("circulation arrest"):ti,ab,kw (Word variations have been searched)

#15 ("circulatory arrest"):ti,ab,kw (Word variations have been searched)

#16 ("heart asystole"):ti,ab,kw (Word variations have been searched)

#17 ("heart standstill"):ti,ab,kw (Word variations have been searched)

#18 ("heart arrest"):ti,ab,kw (Word variations have been searched)

#19 ("recovery of spontaneous circulation"):ti,ab,kw (Word variations have been searched)

#20 ("restoration of spontaneous circulation"):ti,ab,kw (Word variations have been searched)

#21 (ROSC):ti,ab,kw (Word variations have been searched)

#22 ("return of spontaneous circulation"):ti,ab,kw (Word variations have been searched)

#23 ("spontaneous circulation return"):ti,ab,kw (Word variations have been searched)

#24 ("cardio pulmonary resuscitation"):ti,ab,kw (Word variations have been searched)

#25 ("cardiopulmonary resuscitation"):ti,ab,kw (Word variations have been searched)

#26 ("chest compression"):ti,ab,kw (Word variations have been searched)

#27 (reanimation):ti,ab,kw (Word variations have been searched)

#28 (resuscitation):ti,ab,kw (Word variations have been searched)

#29 #7 OR #8 OR #9 OR #10 OR #11 OR #12 OR #13 OR #14 OR #15 OR #16 OR #17 OR #18 OR #19 OR #20 OR #21 OR #22 OR #23 OR #24 OR #25 OR #26 OR #27 OR #28

#30 #6 AND #29

Cinahl

#	Query
S1	(MH "Head+") AND (MH "Oximeters+")
S2	TI (((cerebr* OR brain* OR crani*) N1 (oxigen* OR oxygen* OR oximet* OR oxymet*))) OR AB (((cerebr* OR brain* OR crani*) N1 (oxigen* OR oxygen* OR oximet* OR oxymet*))) OR SU (((cerebr* OR brain* OR crani*) N1 (oxigen* OR oxygen* OR oximet* OR oxymet*)))
S3	TI (((cerebr* OR brain* OR crani*) N2 'near infrared')) OR AB (((cerebr* OR brain* OR crani*) N2 'near infrared')) OR SU (((cerebr* OR brain* OR crani*) N2 'near infrared'))
S4	S1 OR S2 OR S3
S5	(MH "Heart Arrest+")
S6	(MH "Return of Spontaneous Circulation")
S7	(MH "Resuscitation, Cardiopulmonary+")
S8	TI (asystole) OR AB (asystole) OR SU (asystole)
S9	TI (asystolia) OR AB (asystolia) OR SU (asystolia)
S10	TI (asystoly) OR AB (asystoly) OR SU (asystoly)
S11	TI ("cardiac arrest") OR AB ("cardiac arrest") OR SU ("cardiac arrest")
S12	TI ("circulation arrest") OR AB ("circulation arrest") OR SU ("circulation arrest")
S13	TI ("circulatory arrest") OR AB ("circulatory arrest") OR SU ("circulatory arrest")
S14	TI ("heart asystole") OR AB ("heart asystole") OR SU ("heart asystole")
S15	TI ("heart standstill") OR AB ("heart standstill") OR SU ("heart standstill")
S16	TI ("heart arrest") OR AB ("heart arrest") OR SU ("heart arrest")
S17	TI ("recovery of spontaneous circulation") OR AB ("recovery of spontaneous circulation") OR SU ("recovery of spontaneous circulation")
S18	TI ("restoration of spontaneous circulation") OR AB ("restoration of spontaneous circulation") OR SU ("restoration of spontaneous circulation")
S19	TI (ROSC) OR AB (ROSC) OR SU (ROSC)
S20	TI ("return of spontaneous circulation") OR AB ("return of spontaneous circulation") OR SU ("return of spontaneous circulation")
S21	TI ("spontaneous circulation return") OR AB ("spontaneous circulation return") OR SU ("spontaneous circulation return")
S22	TI (CPR) OR AB (CPR) OR SU (CPR)
S23	TI ("cardio pulmonary resuscitation") OR AB ("cardio pulmonary resuscitation") OR SU ("cardio pulmonary resuscitation")
S24	TI ("cardiopulmonary resuscitation") OR AB ("cardiopulmonary resuscitation") OR SU ("cardiopulmonary resuscitation")
S25	TI ("chest compression") OR AB ("chest compression") OR SU ("chest compression")
S26	TI (reanimation) OR AB (reanimation) OR SU (reanimation)
S27	TI (resuscitation) OR AB (resuscitation) OR SU (resuscitation)
S28	S5 OR S6 OR S7 OR S8 OR S9 OR S10 OR S11 OR S12 OR S13 OR S14 OR S15 OR S16 OR S17 OR S18 OR S19 OR S20 OR S21 OR S22 OR S23 OR S24 OR S25 OR S26 OR S27
S29	S4 AND S28

Web of Science

#	Search Query
1	(cerebr* OR brain* OR crani*) NEAR/1 (oxigen* OR oxygen* OR oximet* OR oxymet*) (Topic)
2	(cerebr* OR brain* OR crani*) NEAR/2 "near infrared" (Topic)
3	#2 OR #1
4	"asystole" (Topic)
5	"asystolia" (Topic)
6	"asystoly" (Topic)
7	TS=("cardiac arrest")
8	"circulation arrest" (Topic)
9	"circulatory arrest" (Topic)
10	"heart asystole" (Topic)
11	"heart standstill" (Topic)
12	"heart arrest" (Topic)
13	"recovery of spontaneous circulation" (Topic)
14	"restoration of spontaneous circulation" (Topic)
15	"rosc" (Topic)
16	"return of spontaneous circulation" (Topic)
17	"spontaneous circulation return" (Topic)
18	"cpr" (Topic)
19	"cardio pulmonary resuscitation" (Topic)
20	"cardiopulmonary resuscitation" (Topic)
21	"chest compression" (Topic)
22	"reanimation" (Topic)
23	"resuscitation" (Topic)
24	#23 OR #22 OR #21 OR #20 OR #19 OR #18 OR #17 OR #16 OR #15 OR #14 OR #13 OR #12 OR #11 OR #10 OR #9 OR #8 OR #7 OR #6 OR #5 OR #4
25	#24 AND #3
26	DT=(Editorial Material OR Letter)
27	#25 NOT #26
