# Supplementary material for: Cerebral oxygen monitoring during out-of-hospital cardiac arrest: A scoping review
Source: Resusc Plus. 2025 Sep 3;26:101082. doi: 10.1016/j.resplu.2025.101082 (PMC12744649; doi:10.1016/j.resplu.2025.101082)
Supplement: Supplementary Data 3 [file mmc3.pdf]

Appendix III: Data extraction sheet

The completed data extraction form is accessible through this link:
https://osf.io/wv5ru/?view_only=9b4e605d9a574a14bfb4acbebf0f3466
