# Supplementary material for: Cerebral oxygen monitoring during out-of-hospital cardiac arrest: A scoping review
Source: Resusc Plus. 2025 Sep 3;26:101082. doi: 10.1016/j.resplu.2025.101082 (PMC12744649; doi:10.1016/j.resplu.2025.101082)
Supplement: Supplementary Data 4 [file mmc4.pdf]

Appendix IV: Sources excluded following full-text review

The primary reason for exclusion was prioritized in following order:

1. Retracted studies
2. Wrong source of evidence (e.g. meeting or conference abstracts, reviews, secondary data, trial registrations)
3. Wrong concept
4. Wrong population
5. Wrong setting
6. IHCA and OHCA population not separable
7. Not enough information presented
8. Incomplete translation
9. Duplicates

Retracted studies

Ito N, Nanto S, Nagao K, Hatanaka T, Nishiyama K, Kai T. Regional cerebral oxygen saturation on hospital arrival is a potential novel predictor of neurological outcomes at hospital discharge in patients with out-of-hospital cardiac arrest. Resuscitation. 2012;83:46-50.

Sakaguchi K, Saito S, Takada M, Takahashi K, Onodera Y, Kobayashi T, et al. Correlation between Pulse-Wave Cerebral Tissue Oxygen Saturation and End-Tidal Carbon Dioxide During Cardiopulmonary Resuscitation. Available at SSRN 4873263.

Wrong source of evidence

Allan KS, Drennan IR, Golan E. Re: A pilot study examining the role of regional cerebral oxygen saturation monitoring as a marker of return of spontaneous circulation in shockable (VF/VT) and non-shockable (PEA/Asystole) causes of cardiac arrest. Resuscitation. 2014;85:e121-e2.

Allen CP, Bird JD, Sekhon MS. The dynamic pathophysiology of post cardiac arrest brain injury: “time is brain”. Curr Opin Crit Care. 2025;31:123-130. doi: 10.1097/MCC.0000000000001246.

Ameloot K, Meex I, Genbrugge C, Jans F, Mullens W, Dupont M, et al. The association between global hemodynamics cerebral oxygenation and survival in post-cardiac arrest patients. Anaesthesiology Intensive Therapy. 2014;46:112.

Ameloot K, Meex I, Genbrugge C, Boer W, Jans F, Ferdinande B, et al. Hemodynamic targets during therapeutic hypothermia after cardiac arrest: A prospective observational study. Critical Care. 2015;19:S150.

Bastani A, Lusis E, Wheaton D, J. ONB. The utility of cerebral oximetry as a predictor of neurologic outcome in out-of-hospital cardiac arrest. Ann Emerg Med. 2003;42:S72-S.

Battaglini D, Bogossian EG, Anania P, Premraj L, Cho S-M, Taccone FS, et al. Monitoring of Brain Tissue Oxygen Tension in Cardiac Arrest: a Translational Systematic Review from Experimental to Clinical Evidence. Neurocrit Care. 2024;40:349-63.

Ben-Hamouda N, Oddo M. Monitoring the Brain after Cardiac Arrest: Techniques and Potential Clinical Utility. Medecine Intensive Reanimation. 2019;28:389-97.

Bertini P, Marabotti A, Paternoster G, Landoni G, Sangalli F, Peris A, et al. Regional Cerebral Oxygen Saturation to Predict Favorable Outcome in Extracorporeal Cardiopulmonary Resuscitation: A Systematic Review and Meta-Analysis. J Cardiothorac Vasc Anesth. 2023;37:1265-72.

Bickler P, Feiner J, Rollins M, Meng L. Tissue Oximetry and Clinical Outcomes. Anesth Analg. 2017;124(1):72-82.

Boer C, Duvekot A, Dekker SE, Van Den Brom CE, Viersen VA, De Waard MC. Hyperfibrinolysis is a marker of tissue hypoperfusion in patients with out-of-hospital cardiac arrest. Eur J Anaesthesiol. 2014;31:210.

Bouzat P, Oddo M. Non-invasive cerebral oximetry for the emergent resuscitation of comatose cardiac arrest patients: Is there still some light in the dark? Resuscitation. 2014;85:714-5.

Burrell L, Rice A. Cerebral oximetry monitoring in OHCA. Journal of Paramedic Practice. 2018;10:517-23.

Callaway CW. Cerebral Oximetry and Cardiopulmonary Resuscitation. Journal of the American Heart Association. 2015;4:1-N.PAG.

Chiarini G, Lorusso R, Coco VL, Willers A, Jiritano F. Towards understanding when ECMO is futile. Artif Organs. 2020;44:E106.

Chiarini G, Swol J, Miranda DR, Taccone FS, Belliato M, Delnoij T, et al. Towards understanding when ECMO is futile in cardiac arrest. Perfusion. 2021;36:54.

Chinn E, Wagner BP, Moore JC, Driver B, Puskarich MA. 13 Transesophageal Echocardiography May Improve Cerebral Perfusion Compared to Transthoracic Echocardiography in Out of Hospital Cardiac Arrest. Ann Emerg Med. 2020;76:S6.

Compton S, Ryder A, Medado P, Bastani A, O'Neil BJ. An exploratory analysis to assess the potential role for non-invasive monitoring of cerebral oxygenation in cardiac arrest. Ann Emerg Med. 2006;48:S97-S.

Coppler PJ, Elmer J. Optimizing cerebral oxygen delivery after cardiac arrest: A role for neuromonitoring. Resuscitation. 2021;169:220-2.

Cournoyer A, Chauny J, Iseppon M, Denault A, Cossette S, Notebaert E. Predicting the return of spontaneous circulation using near-infrared spectroscopy monitoring: A systematic review and meta-analysis. Canadian Journal of Emergency Medicine. 2016;18:S44.

Cournoyer A, Chauny JM, Iseppon M, Denault A, Cossette S, Notebaert E. Near-infrared spectroscopy monitoring during cardiac arrest: A systematic review and meta-analysis. Acad Emerg Med. 2016;23:S63-S4.

Cournoyer A, Iseppon M, Chauny JM, Denault A, Cossette S, Notebaert É. Near-infrared Spectroscopy Monitoring During Cardiac Arrest: A Systematic Review and Meta-analysis. Academic emergency medicine : official journal of the Society for Academic Emergency Medicine. 2016;23:851-62.

Cuesta Aguirre, E. J. "Capnografía y oximetría cerebral como herramientas para mejorar la calidad de la reanimación cardiopulmonar y su uso como indicadores predictivos." Nure Investigación. 2024;1–9.

Damian MS, Czosnyka M. Near infrared spectroscopy monitoring--opening a window on the first 24 h after cardiac arrest? Resuscitation. 2014;85:452-3.

Debaty G, Segond N, Crespi C, Sanchez C, Boeuf J, Duhem H, et al. Regional Cerebral Oxygen Saturation to Predict Neurologic Outcome During Out-ofHospital-Cardiac - Arrest: An Ancillary Analysis of a Prospective Interventional Study. Circulation. 2023;148:2.

Drennan I, Gilgan J, Goncharenko K, Lin S. Paramedics using near-infrared spectroscopy in out-of-hospital cardiac arrest: A feasibility study. Canadian Journal of Emergency Medicine. 2018;20:S52.

Eastwood G, Tanaka A, Bellomo R. Cerebral oxygenation in mechanically ventilated cardiac arrest survivors: The impact of hypercapnia. Anaesth Intensive Care. 2016;44:304.

Eertmans W, Genbrugge C, Meex I, Dens J, Jans F, De Deyne C. Continuous cerebral saturation monitoring during therapeutic hypothermia in out-of-hospital cardiac arrest patients. J Neurosurg Anesthesiol. 2016;28:S33.

Eertmans W, Genbrugge C, Meex I, Dens J, Jans F, De Deyne C. The evolution of cerebral oxygen saturation in post-cardiac arrest patients treated with therapeutic hypothermia. Critical Care. 2016;20.

Endo T, Ito N, Nishiyama K, Morooka T, Hayashida K, Beppu S, et al. Late improvement of neurological function after out-of-hospital cardiopulmonary arrest: Incidence prevalence and predictors. Circulation. 2013;128.

Engel TW, Medado P, Thomas C, Wilburn J, Scott T, O'Neil B. End tidal CO2 versus cerebral oximetry for monitoring CPR quality and determination of return of spontaneous circulation. Ann Emerg Med. 2012;60:S77.

Engel T, Thomas CJ, Medado P, Reed B, Millis S, O'Neil BJ. Predicting return of spontaneous circulation using cerebral oximetry and end tidal CO2. Circulation. 2013;128.

Engel T, Thomas C, Scott T, Wilburn J, Medado P, Reed B, et al. Comparison of end tidal CO2 and cerebral oximetry in determining return of spontaneous circulation during cardiopulmonary resuscitation. Ann Emerg Med. 2013;62:S139-S40.

Engel TW, Scott T, Wilburn J, O'Neil B, Medado P, Thomas C. End tidal CO2 vs. cerebral oximetry for monitoring CPR quality. Acad Emerg Med. 2012;19:S264.

Engel T, Thomas C, Medado P, Bastani A, Reed B, Millis S, et al. End tidal CO2 vs cerebral oximetry during cardiopulmonary resuscitation. Resuscitation. 2018;130:e5-e.

Faiver L, Coppler PJ, Ratay C, Elmer J. 386 Association of hypertonic saline treatment with change in cerebral oxygen extraction after cardiac arrest." Resuscitation. 2024; 203: S183–S183.

Faiver L, Coppler PJ, Ratay C, Chae M, Flickinger K, Eun L, et al. Association of hypertonic saline treatment with change in cerebral oxygen extraction after cardiac arrest." Circulation. 2024;150: 2.

Genbrugge C, Boer W, Anseeuw K, Meex I, Jans F, Dens J, et al. Differences in cerebral saturation measured during prehospital advanced life support between patients with presumed cardiac origin and noncardiac origin of cardiac arrest. Critical Care. 2015;19:S152-S3.

Genbrugge C, Boer W, Meex I, Jans F, Deyne C, Dens J. Difference in cerebral saturation during cardiopulmonary resuscitation between survivors with favorable neurological outcome and compromised neurological outcome at hospital discharge. Critical Care. 2015;19:S151.

Genbrugge C, Dens J, Meex I, Boer W, Eertmans W, Sabbe M, et al. Regional Cerebral Oximetry During Cardiopulmonary Resuscitation: Useful or Useless? The Journal of emergency medicine. 2016;50:198-207.

Genbrugge C, Eertmans W, Jans F, Boer W, De Deyne C. Increase in cerebral oxygenation during pre-hospital CPR is associated with ROSC a multicenter study. Circulation. 2016;134.

Genbrugge C, Eertmans W, Salcido DD. Monitor the quality of cardiopulmonary resuscitation in 2020. Curr Opin Crit Care. 2020;26:219-27.

Genbrugge C, Meex I, Jans F, De Deyne C, Boer W, Dens J. Significant difference in increase in cerebral saturation between patients with and without ROSC during prehospital advanced life support. Circulation. 2014;130.

Genbrugge C, Meex I, Scheyltjens S, Dens J, De Deyne C. Start value of cerebral saturation in prehospital cardiac arrest patients: Does it mean something? Critical Care. 2013;17:S116.

Germon TJ, Manara AR, Kane NN, Nelson RJ. Oxygen metabolism during circulatory arrest. J Neurosurg. 1994;81:331-3.

Ghanayem NS, Wernovsky G, Hoffman GM. Near-infrared spectroscopy as a hemodynamic monitor in critical illness. Pediatr Crit Care Med. 2011;12:S27-S32.

Gwinnutt C. Regional cerebral oxygen saturation monitoring during cardiac arrest. Resuscitation. 2014;85:e129.

Hachimi-Idrissi S. Letter to editor: A pilot study examining the role of regional cerebral oxygen saturation monitoring as a marker of return of spontaneous circulation in shockable (VF/VT) and non-shockable (PEA/asystole) cause of cardiac arrest. Resuscitation. 2014;85:e125-e6.

Hayashida K, Suzuki M, Shiroshita-Takeshita A, Ito N, Nishiyama K, Arimoto H, et al. The Product of blood hemoglobin and regional brain oxygen saturation is a simple and excellent predictor for a short-term neurological outcome in patients with post cardiac arrest syndrome. Circulation. 2012;126.

Hayashida K, Suzuki M, Shiroshita-Takeshita A, Honma Y, Kamura H, Hatada T, et al. Relationship between the blood hemoglobin level at hospital arrival and post-cardiac arrest neurological outcome. Circulation. 2012;126.

Higashi H, Yasuda H, Nishiyama K, Minami Y, Hirayama M, Adachi T, et al. Presumption of cardiopulmonary resuscitation for sustaining cerebral oxidation using regional cerebral saturation of oxygen: Observational cohort study (press study). Acad Emerg Med. 2016;23:S249.

Hirose T, Ehara N, Shiozaki T, Wakai A, Nishimura T, Mori N, et al. The relationship between cerebral regional oxygen saturation during extracorporeal cardiopulmonary resuscitation and neurological outcome. Circulation. 2015;132.

Hirose T, Shiozaki T, Nomura J, Katsura K, Ehara N, Wakai A, et al. Effect of prehospital portable monitoring of cerebral regional oxygen saturation in patients with out-of-hospital cardiac arrest. Circulation. 2014;130.

Honzawa H, Yasuda H, Nishiyama K, Ito N, Nagao K, Kimura T, et al. Effect of prehospital advanced care on regional cerebral oxygen saturation at hospital arrival and neurological outcomes at 90 days in out-of-hospital cardiac arrest patients. Acad Emerg Med. 2015;22:S111-S2.

Howells M, Green DW. Cerebral oximetry monitoring during unexpected cardiopulmonary arrest and tension pneumothorax. Eur J Anaesthesiol. 2006;23:266-8.

Huppert EL, Parnia S. Cerebral oximetry: a developing tool for monitoring cerebral oxygenation during cardiopulmonary resuscitation. Ann N Y Acad Sci. 2022;1509:12-22.

Hurlock A, Seeney A, Ranieri N, Burnett R, Douglas R, Majmudar T, et al. Abstract Su1005: Differences in optical metrics of cerebral autoregulation after pediatric cardiac arrest." Circulation. 2024;150.

Inigo-Santiago L, Yang J, Parnia S. The use of continuous cerebral oximetry monitor as an early predictor of neurological outcomes during therapeutic hypothermia after cardiac arrest. Am J Respir Crit Care Med. 2014;189.

Ito N, Kai T, Tsuruoka A, Honma Y, Yasuda H, Mizobuchi M, et al. A multicenter prospective cohort study on a novel neurological prognostic index regional brain oxygen saturation in patients with out-of-hospital cardiac arrest. Circulation. 2012;126.

Ito N, Nishiyama K, Callaway CW, Tsuruoka A, Arimoto H, Morooka T, et al. Noninvasive regional cerebral oxygen saturation is a reliable and readily available neurological prognostic indicator in out-of-hospital cardiac arrest patients: A multicenter prospective cohort study. Circulation. 2012;126:2794.

Ito N, Nanto S, Doi Y, Sawano H, Masuda D, Yamashita S, et al. Regional brain oxygen saturation as a novel index of neurological outcomes in patients with out-of-hospital cardiogenic cardiac arrest. J Am Coll Cardiol. 2010;55:A111.E1040.

Ito N, Nanto S, Doi Y, Kurozumi Y, Hatano M, Tonomura D, et al. Noninvasive Regional Cerebral Oxygen Saturation Can Predict Poor Brain Resuscitation in Patients with Out-of-Hospital Cardiac Arrest. Circulation. 2010;122:2.

Ito N, Nanto S, Nagao K, Hatanaka T, Kai T. Regional cerebral oxygen saturation predicts poor neurological outcome in patients with out-of-hospital cardiac arrest. Resuscitation. 2010;81:1736-7.

Ito N, Nanto S, Nagao K, Hatanaka T, Nishiyama K, Doi Y, et al. Regional cerebral oxygen saturation: A novel index for prompt clinical outcome prediction before starting extracorporeal cardiopulmonary resuscitation in out of hospital cardiac arrest patients. J Am Coll Cardiol. 2011;57:E908.

Ito N, Nanto S, Nagao K, Nishiyama K, Doi Y, Kurozumi Y, et al. Noninvasive regional cerebral oxygen saturation is a novel predictor of neurological outcome in comatose cardiac arrest survivors treated with therapeutic hypothermia. Eur Heart J. 2011;32:350.

Ito N, Nanto S, Nagao K, Nishiyama K, Tsuruoka A, Kurozumi Y, et al. Superiority of a novel index regional brain oxygen saturation for neurological prognostication after out of hospital cardiac arrest. Eur Heart J. 2011;32:190.

Ito N, Nanto S, Nagao K, Hatanaka T, Nishiyama K, Doi Y, et al. Optimal cerebral oxidation levels for brain protection during resuscitation in cases of refractory cardiac arrest. Circulation. 2011;124.

Ito N, Nanto S, Nagao K, Hatanaka T, Nishiyama K, Doi Y, et al. Regional cerebral oxygen saturation as a novel termination of resuscitation rule in cases of refractory out-of-hospital cardiac arrest. Circulation. 2011;124.

Ito N, Nanto S, Nagao K, Hatanaka T, Nishiyama K, Doi Y, et al. Bystander-initiated cardiopulmonary resuscitation can maintain regional cerebral oxygen saturation levels on hospital arrival in patients with refractory out-of-hospital cardiac arrest. Circulation. 2011;124.

Ito N, Nanto S, Nagao K, Hatanaka T, Nishiyama K, Kai T. Bystander-initiated cardiopulmonary resuscitation can curb the deterioration of regional cerebral oxygen saturation on hospital arrival in patients with cardiac arrest. Resuscitation. 2012;83:e167-8.

Jaeger D, Preau S, Girerd N, Lepage X, Chouihed T. 179 Cerebral Near infrared spectroscopy during out-of-hospital cardiac arrest resuscitation to assess neurological outcome: the NISOHCA study protocol." Resuscitation. 2024;203:S92–S92.

Jakkula P, Hästbacka J, Reinikainen M, Loisa P, Tiainen M, Pettilä V, et al. Targeting normoxia vs. mild hyperoxia after cardiac arrest and resuscitation: A randomized pilot trial (NCT02698917). Intensive care medicine experimental. 2018;6.

Jakkula P, Pettilä V, Skrifvars M, Hästbacka J, Loisa P, Tiainen M, et al. Targeting low-normal vs. high-normal mean arterial pressure after cardiac arrest and resuscitation: A randomized pilot trial (NCT02698917). Intensive care medicine experimental. 2018;6.

Jamil M, Thomas C, Engel T, Reed B, Medado P, O'Neil BJ. Trending cerebral oximetry and mean arterial pressure post cardiac arrest to predict neurological outcome. Circulation. 2014;130.

Jehle D, Brader E, Cottington E. Transconjunctival oxygen monitoring as a predictor of cardiac and neurological outcome following resuscitation. Ann Emerg Med. 1985;14(5):496.

Joo WJ, Ide K, Nishiyama K, Ito N, Tanaka H, Tsuchiya J, et al. Prediction of neurologic outcome after extracorporeal cardiopulmonary resuscitation. Crit Care Med. 2019;47.

Kallas HJ, O'Rourke PP, Kallas HJ, O'Rourke PP. Drowning and immersion injuries in children. Curr Opin Pediatr. 1993;5:295-302.

Kano H, Saito T, Bando K, Matsui T, Endo A, Sakurai K, et al. Epinephrine administration (EA) during cardiopulmonary resuscitation (CPR) increases cerebral tissue oxygen saturation: Study with pulse-observing mode of near-infrared spectroscopy. Circulation. 2015;132.

Kano H, Saito T, Matsui T, Endo A, Nagama M, Iwanaga W, et al. Using regional cerebral oxygen saturation measurements to study when to deliver shocks during CPR: Can optimal timing be determined? Circulation. 2014;130.

Kano H, Saito T, Matsui T, Endo A, Nagama M, Iwanaga W, et al. Evaluating the role of continuously monitoring regional cerebral oxygen saturation in predicting the return of spontaneous circulation in cardiac arrest patients: Does the use of an electrocardiogram alone provide adequate information during cardiopulmonary resuscitation? Circulation. 2014;130.

Kawaguchi T, Tsukuda J, Numata K, Hayashi K, Fujitani S. The Role of End-Tidal Carbon Dioxide and Cerebral Oxygen Saturation to Predict Return of Spontaneous Circulation Among Patients With Out-of-Hospital Cardiac Arrest Patients. Ann Emerg Med. 2023;82:S105-S6.

Kawamorita T, Yagi T, Soga T, Hirose H, Watanabe K, Kikushima K, et al. Changes in cerebral oxygen metabolism and hemodynamics during ECPR with therapeutic hypothermia measured by near infrared spectroscopy. Circulation. 2012;126.

KCT0005191. Effect of head elevation during cardiopulmonary resuscitation on cerebral blood flow during cardiac arrest: a pilot study. http://wwwwhoint/trialsearch/Trial2aspx?TrialID=KCT0005191. 2020.

Khan I, Bastani A, Marlow S, Miller V, Zimny E, Neumann J, et al. Cerebral oximetry: Efficacy in predicting adequacy of resuscitation and neurologic outcome in the critically ill. Ann Emerg Med. 2003;42:S55-S6.

Kinoshita K. Possibility of predicting neurological outcome using regional cerebral oxygen saturation (rSO2) after cardiac arrest...Resuscitation. 2014 Jun;85(6):778-84. Resuscitation. 2014;85:e133-e.

Kool M, Atkins DL, Van de Voorde P, Maconochie IK, Scholefield BR. Focused echocardiography end-tidal carbon dioxide arterial blood pressure or near-infrared spectroscopy monitoring during paediatric cardiopulmonary resuscitation: A scoping review. Resuscitation plus. 2021;6:100109.

Kurth CD, Levy W, Chance B. Oxygen metabolism during circulatory arrest. J Neurosurg. 1994;81:330-1.

Larrea A, Salaberria R, Alonso D, Irusta U, Aramendi E, Isasi I, et al. Monitoring chest compression rate using cerebral oximetry. Resuscitation. 2018;130:e114-e5.

Larrea A, Salaberria R, Amas L, Alonso D, Blanco U, Casas J, et al. Haemoglobin concentration measured by high time resolution cerebral oximetry can monitor chest compression frequency in out-of-hospital cardiac arrest. Resuscitation. 2019;142:e85-e6.

Lathouwers K, De Sloovere V, Jans F, Heylen R, De Deyne C. New technology of noninvasive cerebral oximetry (FORE-SIGHT technology) to monitor cerebral perfusion during resuscitation from cardiac arrest. J Neurosurg Anesthesiol. 2010;22:423.

Laurikkala J, Aneman A, Peng A, Reinikainen M, Pham P, Jakkula P, et al. Association of deranged cerebrovascular autoregulation with brain injury following cardiac arrest: A post hoc analysis of the COMACARE trial. Intensive care medicine experimental. 2021;9.

Lee B, et al. "Cerebral oxygen saturation is associated with neurological outcomes in cardiac arrest survivors. Critical care medicine. 2025;53: 1.

Levy WJ, Levin S. CEREBRAL OXYGEN-SATURATION DURING CARDIAC-ARREST. Anesthesiology. 1994;81:A533.

Lim SL, Chan SP, Woo KL, Chua ZX, Lee R, Shahidah N, et al. Regional cerebral oxygen saturation in resuscitated cardiac arrest patients. Resuscitation. 2023;192:S50-S1.

Liu Y, Jing K, Liu H, Mu Y, Jiang Z, Nie Y, et al. Association between cerebral oximetry and return of spontaneous circulation following cardiac arrest: A systematic review and meta-analysis. PloS one. 2020;15.

Long B, Gottlieb M. Emergency medicine updates: Cardiopulmonary resuscitation. Am J Emerg Med. 2025:93:86-93. doi: 10.1016/j.ajem.2025.03.057.

Magallanes C. Cerebral Oximetry During Cardiac Arrest: A Multicenter Study of Neurologic Outcomes and Survival: Parnia S Yang J Nguyen R et al. Crit Care Med. 2016;44:1663-1674. Journal of Emergency Medicine (0736-4679). 2016;51:614-.

Major R, Starr Z, Aziz S, Lachowycz K, Hill L. OP06 Is intra-arrest cerebral oxygen saturation associated with return of spontaneous circulation in out-of-hospital cardiac arrest patients? – A prospective feasibility study. Emergency Medicine Journal. 2024;41: A4.1-A4

Marabotti A, Guarracino F, Bertini P. Regional cerebral oxygen saturation as an outcome-predicting marker for ECPR recipients: A meta-analysis. Perfusion. 2022;37:8.

Marquez AM, Morgan RW, Ross CE, Berg RA, Sutton RM. Physiology-directed cardiopulmonary resuscitation: advances in precision monitoring during cardiac arrest. Current opinion in critical care. 2018;24:143-50.

Mayr NP, Martin K, Hausleiter J, Tassani P. Measuring cerebral oxygenation helps optimizing post-resuscitation therapy...Resuscitation. 2010 Dec;81(12):1736-7. Resuscitation. 2011;82:1110-1.

Mayr NP, Martin K, Kurz J, Tassani P. Monitoring of cerebral oxygen saturation during closed-chest and open-chest CPR. Resuscitation. 2011;82:635-6.

Medado P, Thomas CJ, Scott T, Kessler J, O'Neil B. Cerebral oximetry as a post-ROSC predictor of neurologic outcome after OOHCA. Circulation. 2012;126.

Medicherla CB, Lewis A. The critically ill brain after cardiac arrest. Annals of the New York Academy of Sciences. 2022;1507:12-22.

Meex I, Dens J, Jans F, De Deyne C. Can cerebral oxygenation after cardiac arrest be correlated to outcome? European journal of anaesthesiology. 2012;29:S8.

Meex I, Dens J, Jans F, De Deyne C. NIRS cerebral oxygenation monitoring during induction of therapeutic hypothermia after cardiac arrest. European journal of anaesthesiology. 2012;29:54.

Meex I, Dens J, Jans F, De Deyne C. Is there a relation between cerebral oxygenation after cardiac arrest and outcome? European journal of anaesthesiology. 2012;29:49.

Meex IM, De Deyne C, Dens J, Jans F, Lathouwers K, Heylen R. Shivering during induced hypothermia after cardiac arrest results in significant decreases in noninvasive cerebral oxygenation. Journal of neurosurgical anesthesiology. 2011;23:391-2.

Meex IM, De Deyne C, Jans F, Dens J, Lathouwers K, Heylen R. Cerebral oxygenation measured by nirs fore-sight technology reveals adequacy of cerebral perfusion during cardiopulmonary resuscitation. Journal of neurosurgical anesthesiology. 2011;23:390-1.

Moore JC, Fagerstrom ET, Robinson A, Boland J, Harrington J, Driver B, et al. The use of regional cerebral oxygen and tissue oxygenation monitoring during and immediately

after cardiac arrest in the emergency department. Academic Emergency Medicine. 2016;23:S205.

Nakahori Y, Shimizu K, Shiozaki T, Ohnisi M, Nakagawa Y, Tasaki O, et al. Assessment of the Benefit of Extracorporeal Life-Support for Patients With Cardiac Arrest in Terms of Cerebral Oxygen Saturation. Circulation. 2010;122:2.

Nakahori Y, Shimizu K, Shiozaki T, Ogura H, Tasaki O, Kuwagata Y. The change of cerebral rSO2 during cardiopulmonary resuscitation. Crit Care Med. 2008;36:A152.

Nakatani Y, Nakayama T, Nishiyama K, Takahashi Y. Effect of target temperature management at 32-34 °c in cardiac arrest patients stratified by regional cerebral oxygen saturation: A multicenter retrospective cohort study in Japan. Resuscitation. 2017;118:e27-e8.

Impact of NIRS-guided Cardiopulmonary Resuscitation After Cardiac Arrest on Resuscitation Rate. https://clinicaltrialsgov/show/NCT03911908. 2019.

PERSEUS-PS Randomized Controlled Trial. https://clinicaltrialsgov/show/NCT04428060. 2020.

Nelskyla A, Skrifvars M, Ngerman SA, Nurmi J. Arterial oxygen and cerebral oxygenation during clinical cardiopulmonary resuscitation. Intensive care medicine experimental. 2021;9.

Niemelae VH, Reinikainen M, Nielsen N, Bass F, Young P, Lilja G, et al. Higher versus lower mean arterial blood pressure after cardiac arrest and resuscitation (MAP-CARE): A protocol for a randomized clinical trial. Acta Anaesthesiol Scand. 2025;69:e70040. doi: 10.1111/aas.70040.

Nishioka N, Kiguchi T, Makino Y, Ninomiya K, Kamo W, Kamada T, et al. Association between Increment of Near-Infrared Spectroscopy-Derived Cerebral Perfusion Index and Return of Spontaneous Circulation in Out-of-Hospital Cardiac Arrest Patients: A Prospective Observational Study. Circulation. 2024;150: 2.

Nishiyama K, Hamanaka K, Yoshida K, Tsuchiya J, Murakami H, Kawaguchi R, et al. Brain regional oxygen saturation monitoring by emergency medical stuff for goal-directed resuscitation. Resuscitation. 2018;130:e113-e.

Nishiyama K, Ito N, Abe M, Unoki T, Endo T, Himeno H, et al. Severity assessment of brain damage with RSO2 monitoring for decisions regarding intensive care after out-of-hospital cardiac arrest with presumed cardiac causes. Circulation. 2014;130.

Nishiyama K, Ito N, Orita T, Abe M, Yasuda H, Honma Y, et al. Characteristics of regional cerebral oxygen saturation levels in patients who experience out-of-hospital cardiac arrest with or without return of spontaneous circulation. European heart journal. 2015;36:1196.

Nishiyama K, Ito N, Otsuka M, Morooka T, Abe M, Orita T, et al. Sex-related differences of cerebral circumstances and clinical outcomes in patients with out-of-hospital cardiopulmonary arrest. European heart journal. 2013;34:728.

Nishiyama K, Ito N, Tsuruoka A, Hayashida K, Takabayashi T, Yamada N, et al. Serial characterization of neurological function after out-of-hospital cardiopulmonary arrest: Incidence prevalence and predictors of late-improvement. European heart journal. 2013;34:577.

Nishiyama K, Ueda T, Suitsu Y, Hamanaka K, Tanaka H, Shimoto M, et al. Utility of brain regional oxygen saturation monitoring in out-of-hospital cardiac arrest severity and quality cardio pulmonary resuscitation evaluation: A prospective observational cohort study. European Heart Journal: Acute Cardiovascular Care. 2018;7:326-7.

Nolan JP. Cerebral oximetry during cardiac arrest-feasible but benefit yet to be determined*. Critical care medicine. 2014;42:1001-2.

O'Neil BJ. Prediction of return of spontaneous circulation: Comparison of cerebral oximetry and end tidal CO2. Circulation. 2018;138.

O'Neill BJ, Mangona V, Medado P, Ryder A, Robinson D, Swor R, et al. Cerebral oximetry as an indicator of cerebral autoregulaton in out-of-hospital cardiac arrest patients. Annals of emergency medicine. 2007;50:S60-S.

Ogawa Y. Load-distributing-band CPR for cardiac arrest patients improves regional cerebral oxygen saturation. Circulation. 2013;128.

Ogawa Y, Shiozaki T, Hirose T, Ohnishi M, Ogura H, Shimazu T, et al. The rSO2 value is useful as a new index of ROSC. Circulation. 2014;130.

Ogawa Y, Shiozaki T, Hirose T, Ohnishi M, Tajima G, Nishimura T, et al. The monitoring of rSO2 value during CPR is useful for high-quality CPR. Circulation. 2015;132.

Orita T. The potential usefulness of regional cerebral oxygen saturation (RSO2) monitoring for better cardiopulmonary cerebral resuscitation (CPCR) and post-cardiac arrest care. Shock. 2016;46:69.

Orita T, Toyoda Y, Nakamichi Y, Yamazaki M, Funabiki T, Shimizu M, et al. The efficacy of real-time monitoring by regional cerebral oxygen saturation during chest compression cardiopulmonary resuscitation for avoiding poor neurological outcome. Circulation. 2014;130.

Orita T, Toyoda Y, Yamazaki M, Funabiki T, Shimizu M, Matsumoto S, et al. The possibility of real-time monitoring by regional cerebral oxygen saturation (RSO2) as an indicator of quality of chest compression cardiopulmonary resuscitation for out-of-hospital cardiac arrest patients. Circulation. 2012;126.

Papadimos TJ, Marco AP. Cerebral oximetry and an unanticipated circulatory arrest. Anaesthesia. 2004;59:309-10.

Parnia S. Cerebral oximetry - The holy grail of non-invasive cerebral perfusion monitoring in cardiac arrest or just a false dawn? Resuscitation. 2012;83:11-2.

Parnia S, Yang J, Inigo-Santiago L, Ahn A, Zhu JW, Nasir A, et al. Cerebral Oximetry is a Predictor of Return of Spontaneous Circulation in Cardiac Arrest. Circulation. 2014;130:2124-.

Parnia, S. Physiologically Guided Cardiopulmonary Resuscitation Using End Tidal Carbon Dioxide and Cerebral Oximetry: A Multi-Site Observation Study. Circulation. 2023; 148: E313–E313.

Petrovčič R, Rakusa M, Markota A. Monitoring of Cerebral Blood Flow Autoregulation after Cardiac Arrest. Medicina (Kaunas, Lithuania). 2024;60(9).

Price Y, Anwar S. Response to Use of Cerebral Oximetry During Cardiac Arrest. Critical Care Medicine. 2017;45:e334-e5.

Qiu C, Shah NK. Cerebral oximetry. Progress in Anesthesiology. 2001;15(24):423-37.

Ramakers F, De Deyne C, Jans F, Vandermeersch E, Heylen R. New technology of non-invasive cerebral oximetry (Fore-Sight technology) to monitor cerebral perfusion during resuscitation from cardiac arrest (CPR). European journal of anaesthesiology. 2010;27:60-.

Ravishankar S, Yang J, Zhu J, Nguyen R, Patel JK, Schoenfeld E, et al. Cerebral oximetry: A predictor of neurological outcome after cardiac arrest. American Journal of Respiratory and Critical Care Medicine. 2015;191.

Reagan EM, Nguyen RT, Ravishankar S, Chabra V, Fuentes B, Spiegel R, et al. The association between cerebral oxygenation and brain waves measured by cerebral oximetry and electroencephalography (EEG) during cardiopulmonary resuscitation (CPR): A feasibility study. Circulation. 2015;132.

Redfors B, Byttner A, Bengtsson D, Watson P, Lundgren P, Gäbel J, et al. Selection of patients for extra corporeal cardiopulmonary resuscitation (ECPR) - a multivariate prediction model for good neurological outcome. Perfusion. 2023;38:112.

Reis C, Akyol O, Araujo C, Huang L, Enkhjargal B, Malaguit J, et al. Pathophysiology and the Monitoring Methods for Cardiac Arrest Associated Brain Injury. International journal of molecular sciences. 2017;18:18.

Sakaguchi K, Saito S, Takada M, Takahashi K, Onodera Y, Kobayashi T, et al. The relation between cerebral tissue oxygen saturation and end tidal carbon dioxide during cardiopulmonary resuscitation. Intensive care medicine experimental. 2020;8.

Sakaguchi K, Takada S, Takada M, Takahashi K, Onodera Y, Kobayashi T, et al. Is NIRO Pulse a useful monitor for cardiopulmonary resuscitation? Intensive care medicine experimental. 2018;6.

Sakai T, Shiozaki T, Ohnishi M, Takegawa R, Hirose T, Shimazu T. Is cerebral regional oxygen saturation (rSO2) useful as a physiological monitoring tool during CPR in pre-hospital settings?: An analysis of 51 cases in Osaka Japan. Circulation. 2017;136.

Sakai T, Shiozaki T, Ohnishi M, Takegawa R, Tachino J, Shimazu T. Is cerebral regional oxygen saturation (rSO2) useful as a physiological monitoring tool during CPR in pre-hospital settings? An analysis of 72 cases in Osaka Japan. Circulation. 2018;138.

Salaberria R, Larrea A, Alonso D, Aramendi E, Irusta U, Ortega JI, et al. Evaluation of the increase in cerebral oximeter saturation during out-of-hospital mechanical chest compression sequences. Resuscitation. 2018;130:e105-e.

Salaberria R, Larrea A, Amas L, Alonso D, Etxeberria N, Egibar I, et al. Cerebral oximetry vs capnography to monitor chest compressions and return of spontaneous circulation in out-of-hospital cardiac arrest. Resuscitation. 2019;142:e88-e9.

Salaberria R, Larrea A, Amas L, Zubia F, Zubero MB, Alonso D, et al. Cerebral Oximetry to Identify Return of Spontaneous Circulation in Patients Treated With Manual or Mechanical Chest Compressions. Circulation. 2019;140.

Salaberria R, Redin AL, Aramendi E, Cabezas Z, Mugica EM, Arnedo A. Cerebral Oximetry Shows The Cardiopulmonary Resuscitation Leading To Return Of Spontaneous Circulation. Circulation. 2022;146.

Salwey O, Franklin D, Hughes A, Marsden M. REBOA in non-traumatic cardiac arrest: a review of the literature. Emergency Medicine Journal. 2024; 41: 25–26.

Sandroni C, Parnia S, Nolan JP. Cerebral oximetry in cardiac arrest: a potential role but with limitations. Intensive care medicine. 2019;45:904-6.

Sanfilippo F, La Via L, Dezio V, Astuto M, Morgana A. Monitoring of cerebral oxygenation during cardiopulmonary resuscitation may dramatically reduce the incidence of severe hyperoxia. Resuscitation. 2022;170:363-4.

Sanfilippo F, La Via L, Tigano S, Astuto M. Establishing the role of cerebral oximetry during cardio-pulmonary resuscitation of cardiac arrest patients. Resuscitation. 2021;164:1-3.

Sanfilippo F, Murabito P, Messina A, Dezio V, Busalacchi D, Ristagno G, et al. Cerebral regional oxygen saturation during cardiopulmonary resuscitation and return of spontaneous circulation: A systematic review and meta-analysis. Resuscitation. 2021;159:19-27.

Sanfilippo F, Serena G, Corredor C, Benedetto U, Maybauer MO, Al-Subaie N, et al. Cerebral oximetry and return of spontaneous circulation after cardiac arrest: A systematic review and meta-analysis. Resuscitation. 2015;94:67-72.

Schnaubelt S, Mayr F, Sterz F, Zajicek A, Uray T. Feasibility of continuous NIRS monitoring during out-of hospital cardiac arrest until 72 hours post-ROSC. Resuscitation. 2019;142:e10.

Schnaubelt S, Sulzgruber P, Menger J, Skhirtladze-Dworschak K, Sterz F, Dworschak M. Regional cerebral oxygen saturation during cardiopulmonary resuscitation as a predictor of return of spontaneous circulation and favourable neurological outcome – A review of the current literature. Resuscitation. 2018;125:39-47.

Schoonackers J, Van de Voorde P. Cerebral tissue oximetry in cardiopulmonary resuscitation: a systematic review of the literature. Acta Clinica Belgica. 2021;76:16-7.

Scott TM, Cloyd J, Wilburn J, Kessler J, Engle T, Thomas C, et al. Using cerebral oxiemtry to predict return of spontaneous circulation. Annals of emergency medicine. 2012;60:S11.

Shao R., Hang C, Wang X, Zhang L, Shao F, Tang Z. The "SOOTEST-ICU" bundle for optimizing cerebral hypoxia and reperfusion to minimize brain injury after resuscitation from cardiac arrest. World J Emerg Med. 2025;16:206–211. doi: 10.5847/wjem.j.1920-8642.2025.050

Sharma V. Efficacy of Neuroinvasive Goal-Directed Therapy in Hypoxic Ischemic Brain Injury After Cardiac Arrest. Critical Care Alert. 2022;29:1-3.

Shigematsu S, Sakai T, Shiozaki T, Ohnishi M, Takegawa R, Tachino J, et al. Cerebral Regional Oxygen Saturation (rSO2) is Useful as a Physiological Monitoring Tool During CPR in the Pre-Hospital Settings. An Analysis of 87 Cases in Osaka Japan. Circulation. 2019;140.

Shin J, Blackwood JE, Walker RG, Chapman FW, Crackel J, Rea T. Cerebral Oximetry During Resuscitation: Course and Outcome. Circulation. 2019;140.

Singer AJ, Ahn A, Thode JHC, Henry MC, Parnia S. Cerebral oximetry monitoring during CPR is associated with return of spontaneous circulation but not survival in ED patients in cardiac arrest. Academic Emergency Medicine. 2013;20:S220.

Singer AJ, Nguyen RT, Ravishankar ST, Schoenfeld ER, Parnia S. Cerebral oximetry versus end tidal Co2 in predicting ROSC after cardiac arrest. Academic Emergency Medicine. 2015;22:S111.

Skhirtladze-Dworschak K, Dworschak M. Cerebral oximetry and cardiac arrest. Seminars in cardiothoracic and vascular anesthesia. 2013;17:269-75.

Slovis JC, Bach A, Beaulieu F, Zuckerberg G, Topjian A, Kirschen MP. Neuromonitoring after Pediatric Cardiac Arrest: Cerebral Physiology and Injury Stratification. Neurocritical care. 2024;40:99-115.

Taccone FS, Vincent J-L, de Backer D. Cerebral oximetry to adjust cerebral and systemic circulation after cardiac arrest. Intensive Care Medicine. 2013;39:970-1.

Tachino J, Shiozaki T, Takegawa R, Sakai T, Nakao S, Ohnishi M, et al. Analysis of Neurological Prognosis After Cardiac Arrest Patients by Using Cerebral Regional Oxygen Saturation Monitoring. Circulation. 2019;140.

Tajima G, Shiozaki T, Izumino H, Yamano S, Inokuma T, Hirao T, et al. Monitoring system of regional cerebral oxygen saturation during pre-hospital cardiopulmonary resuscitation. Circulation. 2013;128.

Tajima G, Shiozaki T, Ogawa Y, Hirose T, Mori N, Ueki T, et al. Differential recovery between regional cerebral oxygen saturation and physiological parameters in cardiopulmonary arrest patients after return of spontaneous circulation. Circulation. 2015;132.

Tajima G, Shiozaki T, Ogawa Y, Hirose T, Mori N, Ueki T, et al. Correlation between regional cerebral oxygen saturation (RSO2) and arterial blood gas (ABG) during cardiopulmonary resuscitation. Circulation. 2016;134.

Takegawa R, Hayashida K, Rolston DM, Li T, Miyara SJ, Ohnishi M, et al. Near-Infrared Spectroscopy Assessments of Regional Cerebral Oxygen Saturation for the Prediction of Clinical Outcomes in Patients With Cardiac Arrest: A Review of Clinical Impact Evolution and Future Directions. Frontiers in medicine. 2020;7:10.

Takegawa R, Shiozaki T, Hirose T, Mori N, Sakai T, Ohnishi M, et al. Can we predict the probability of ROSC by the change in cerebral RSO2 value? Circulation. 2016;134.

Takegawa R, Shiozaki T, Ohnishi M, Muratsu A, Tachino J, Sakai T, et al. The TripleCPR 16 Study: Does Rhythm Truly Needed to Be Checked Every 2 Minutes in Cardiopulmonary Arrest Patients? Circulation. 2019;140.

Takegawa R, Shiozaki T, Ohnishi M, Tachino J, Muroya T, Sakai T, et al. The triple CPR 16 study: Does rhythm truly. Circulation. 2018;138.

Tamura T, Ito N, Nishiyama K, Hayashida K, Suzuki M, Mochizuki T, et al. Sex difference of clinical outcomes in patients with out-of-hospital cardiopulmonary arrest: A prospective multicenter observational study. Circulation. 2013;128.

Thomas C, Engel T, Reed B, Medado P, Millis S, O'Neil B. Cerebral oximetry and end tidal CO2 as predictors of futility during cardiopulmonary resuscitation. Circulation. 2013;128.

Tobias JD. Cerebral oxygenation monitoring: near-infrared spectroscopy. Expert Review of Medical Devices. 2006;3:235-43.

Tsukuda J, Kurisu M, Kawaguchi T, Takamatsu Y, Yanai M, Morisawa K, et al. Effectiveness of saturation of brain tissue as a prognosis predictive value in CPA. Critical care medicine. 2016;44:147.

Unoki T, Nishiyama K, Ito N, Orita T, Arimoto H, Beppu S, et al. Severity assessment of brain damage with RSO2 monitoring for extracorporeal cardiopulmonary resuscitation after out-of-hospital cardiac arrest. Circulation. 2016;134.

Unoki T, Nishiyama K, Ito N, Tsujimura Y, Abe M, Beppu S, et al. Patients with moderate cerebral perfusion deficit upon hospital arrival may be good candidates for therapeutic hypothermia following out-of-hospital cardiac arrest. European heart journal. 2015;36:577.

Van den Bempt S, Wauters L, Dewolf P. Pulseless Electrical Activity: Detection of Underlying Causes in a Prehospital Setting. Medical Principles & Practice. 2021;30:212-22.

Wesley K, Wesley K. CEREBRAL OXYGENATION. A new cardiac arrest correlation lacks implication. JEMS : a journal of emergency medical services. 2015;40:25.

Wik L. Near-infrared spectroscopy during cardiopulmonary resuscitation and after restoration of spontaneous circulation: a valid technology? Current opinion in critical care. 2016;22:191-8.

Wilburn JM, Cloyd J, O'Neil B, Medado P, Scott T, Engel T. Comparison of emergency department and out-of- hospital cardiac arrest patients monitored with end tidal CO2 and cerebral oximetry. Annals of emergency medicine. 2012;60:S76-S7.

Xiang P, L. X. Application of Near Infrared Spectroscopy in Cardiopulmonary Resuscitation. Medical Journal of Peking Union Medical College Hospital. 2023;14:459-64.

Yasuda H. Evaluation of the quality of chest compressions using near infrared spectroscopy sensor. Critical Care medicine. 2016;44:169.

Yasuda H. Presumption of cardiopulmonary resuscitation for sustaining cerebral oxidation using regional cerebral saturation of oxygen: Observational cohort study (press study). Critical Care. 2016;20.

Yasuda H, Nishiyama K, Ito N, Arimoto H, Orita T, Akira M, et al. Impact of prehospital advanced care on regional brain oxygen saturation at hospital arrival and neurological outcomes at 90 days in patients with out-of-hospital cardiopulmonary arrest. Circulation. 2014;130.

Wrong concept

Brodersen P. Cerebral blood flow and metabolism in coma following cardiac arrest. Revue d'EEG et de Neuro-Physiologie Clinique. 1974;4:329-33.

Frisch A, Suffoletto BP, Frank R, Martin-Gill C, Menegazzi JJ. Potential utility of near-infrared spectroscopy in out-of-hospital cardiac arrest: an illustrative case series. Prehosp Emerg Care. 2012;16(4):564-70.

Kim DW, Choi JK, Won SH, Yun YJ, Jo YH, Park SM, et al. A new variant position of head-up CPR may be associated with improvement in the measurements of cranial near-infrared spectroscopy suggestive of an increase in cerebral blood flow in non-traumatic out-of-hospital cardiac arrest patients: A prospective interventional pilot study. Resuscitation. 2022;175:159-66.

Mori N, Hirose T, Shiozaki T, Ogawa Y, Takegawa R, Tachino J, et al. Effectiveness of the hemoglobin index for screening of subarachnoid hemorrhage in out-of-hospital cardiopulmonary arrest patients: a retrospective observational study. Acute medicine & surgery. 2020;7:6.

Morris NA, Robinson D, Schmidt JM, Frey HP, Park S, Agarwal S, et al. Hunt-Hess 5 subarachnoid haemorrhage presenting with cardiac arrest is associated with larger volume bleeds. Resuscitation. 2018;123:71-6.

Müllner M, Sterz F, Domanovits H, Zeiner A, Laggner AN. Systemic and cerebral oxygen extraction after human cardiac arrest. European journal of emergency medicine : official journal of the European Society for Emergency Medicine. 1996;3:19-24.

Wrong population

Chatzopoulou G, Voucharas C, Kougkouli I, Stathoulopoulos, Linardatou V. Comparison of Clinical Outcomes Between Alpha-Stat and pH-Stat Strategies During Hypothermic Circulatory Arrest: A Systematic Review. Cureus. 2025;17:e87279. doi: 10.7759/cureus.87279.

Devine, K, Parnia K, Gonzales A. Should Packed Red Blood Cells Be Used to Improve Oxygen Delivery Beyond Cardiopulmonary Resuscitation? A Case Study. American Journal of Respiratory and Critical Care Medicine. 2025; 211.

Faiver L, Coppler PJ, Tam J, Ratay CR. Association of hyperosmolar therapy with cerebral oxygen extraction after cardiac arrest. Resuscitation. 2024.

Kaya FB, Acar N, Ozakin E, Canakci ME, Kuas C, Bilgin M. Comparison of manual and mechanical chest compression techniques using cerebral oximetry in witnessed cardiac arrests at the emergency department: A prospective randomized clinical study. Am J Emerg Med. 2021;41:163-9.

Koyama Y, Mizutani T, Marushima A, Sonobe A, Shimojo N, Kawano S. Cerebral tissue oxygenation index using near-infrared spectroscopy during extracorporeal cardio-pulmonary resuscitation predicted good neurological recovery in a patient with acute severe anemia. Intern Med. 2017;56:2451-3.

McCormick PW, Balakrishnan G, Stewart M, Lewis G, Ausman JI. Cerebral oxygen metabolism measured during hypothermic circulatory arrest: A case report. Journal of neurosurgical anesthesiology. 1991;3:302-7.

Renz D, Kraus B, Karliczek GF. Regional cerebral oxygen saturation in hypothermic cardiopulmonary arrest. Anasthesiologie Intensivmedizin Notfallmedizin Schmerztherapie Supplement. 1997;32:S231-S4.

Wrong setting

Ameloot K, Meex I, Genbrugge C, Jans F, Boer W, Verhaert D, et al. Hemodynamic targets during therapeutic hypothermia after cardiac arrest: A prospective observational study. Resuscitation. 2015;91:56-62.

Belohlavek J, Skalicka H, Boucek T, Kovarnik T, Fichtl J, Smid O, et al. Feasibility of cerebral blood flow and oxygenation monitoring by continuous transcranial Doppler combined with cerebral oximetry in a patient with refractory cardiac arrest treated by extracorporeal life support. Perfusion (United Kingdom). 2014;29:534-8.

Bouglé A, Daviaud F, Bougouin W, Rodrigues A, Geri G, Morichau-Beauchant T, et al. Determinants and significance of cerebral oximetry after cardiac arrest: A prospective cohort study. Resuscitation. 2016;99:1-6.

Bouzat P, Suys T, Sala N, Oddo M. Effect of moderate hyperventilation and induced hypertension on cerebral tissue oxygenation after cardiac arrest and therapeutic hypothermia. Resuscitation. 2013;84:1540-5.

Eastwood GM, Tanaka A, Bellomo R. Cerebral oxygenation in mechanically ventilated early cardiac arrest survivors: The impact of hypercapnia. Resuscitation. 2016;102:11-6.

Gomersall CD, Joynt GM, Gin T, Freebairn RC, Stewart LET. Failure of the INVOS 3100 cerebral oximeter to detect complete absence of cerebral blood flow. Crit Care Med. 1997;25:1252-4.

Inal MT, Memiş D, Yıldırım I, Uğur H, Erkaymaz A, Turan FN. The prognostic value of cerebral oxygen saturation measurement for assessing prognosis after cardiopulmonary resuscitation. Brazilian Journal of Anesthesiology. 2017;67:355-61.

Kim TJ, Kim JM, Lee JS, Park SH, Jeong HB, Choi JK, et al. Prognostication of neurological outcome after cardiac arrest using wavelet phase coherence analysis of cerebral oxygen. Resuscitation. 2020;150:41-9.

Kirschen MP, Majmudar T, Beaulieu F, Burnett R, Shaik M, Morgan RW, et al. Deviations from NIRS-derived optimal blood pressure are associated with worse outcomes after pediatric cardiac arrest. Resuscitation. 2021;168:110-8.

Laurikkala J, Aneman A, Peng A, Reinikainen M, Pham P, Jakkula P, et al. Association of deranged cerebrovascular reactivity with brain injury following cardiac arrest: a post-hoc analysis of the COMACARE trial. Critical Care. 2021;25.

Lim SL, Myint MZ, Woo KL, Chee EYH, Hong CS, Beqiri E, et al. Multi-Modal Assessment of Cerebral Hemodynamics in Resuscitated Out-of-Hospital Cardiac Arrest Patients: A Case-Series. Life (Basel). 2024;14:1067. doi: 10.3390/life14091067.

Ryu SJ, Lee BK, Lee DH, Kim DK, Cho YS, Lee JH, et al. The association between regional cerebral oxygen saturation and neurological outcomes in cardiac arrest survivors. Medicine (Baltimore). 2025;104:e42992.

Sakurai A, Ihara S, Tagami R, Yamaguchi J, Sugita A, Kuwana T, et al. Parameters Influencing Brain Oxygen Measurement by Regional Oxygen Saturation in Postcardiac Arrest Patients with Targeted Temperature Management. Therapeutic hypothermia and temperature management. 2020;10:71-5.

Yazar MA, Kozanhan B, Tire Y, Sekmenli N, Yazar G, Sevim M. Combined sonographic optic nerve sheath diameter and cerebral oximeter for predicting neurological outcome after cardiac arrest. Biomol Biomed. 2025;25:672-681. doi: 10.17305/bb.2024.11442.

IHCA and OHCA population not separable

Abramo TJ, Meredith M, Jaeger M, Schneider B, Bagwell H, Ocal E, et al. Cerebral oximetry with blood volume index in asystolic pediatric cerebrospinal fluid malfunctioning shunt patients. Am J Emerg Med. 2014;32:1439.e1-.e7.

Ahn A, Nasir A, Malik H, D'Orazi F, Parnia S. A pilot study examining the role of regional cerebral oxygen saturation monitoring as a marker of return of spontaneous circulation in shockable (VF/VT) and non-shockable (PEA/Asystole) causes of cardiac arrest. Resuscitation. 2013;84:1713-6.

Engel TW, Thomas C, Medado P, Bastani A, Reed B, Millis S, et al. End tidal CO2 and cerebral oximetry for the prediction of return of spontaneous circulation during cardiopulmonary resuscitation. Resuscitation. 2019;139:174-81.

Francoeur C, Landis WP, Winters M, Naim MY, Donoghue A, Dominick CL, et al. Near-infrared spectroscopy during cardiopulmonary resuscitation for pediatric cardiac arrest: A prospective observational study. Resuscitation. 2022;174:35-41.

Reagan EM, Nguyen RT, Ravishankar ST, Chabra V, Fuentes B, Spiegel R, et al. Monitoring the relationship between changes in cerebral oxygenation and electroencephalography patterns during cardiopulmonary resuscitation: A feasibility study. Critical care medicine. 2018;46:757-63.

Not enough information presented

Sakaguchi K, Saito S, Takada M, Taskahashi K, Onodera Y, Kobayashi T, et al. Correlation between pulse-wave cerebral tissue oxygen saturation and end-tidal carbon dioxide during cardiopulmonary resuscitation. Am J Emerg Med. 2025:96:134-139. doi: 10.1016/j.ajem.2025.06.051.

Sanz-Pescador A, Alonso E, Isasi I, Larrea A, Salaberria R, Aramendi. Monitoring Chest Compression Rate in Cerebral Oximetry Signals during Cardiopulmonary Resuscitation using Wavelet Analysis. Annu Int Conf IEEE Eng Med Biol Soc. 2024:2024:1-4. doi: 10.1109/EMBC53108.2024.10781526.

Ångerman S, Länkimäki S, Neuvonen N, Kirves H, Nurmi J. Prospective pilot study of cerebral near infrared spectroscopy monitoring during pre-hospital anaesthesia. Acta Anaesthesiol Scand. 2018;62:1139-45.

Incomplete translation

Nagama M, Kano H, Yasutake Y, Ohira S, Onishi H, Mukai M, et al. A case of cardiac arrest due to diabetic ketoacidosis with blood glucose of 2,105mg/dL was saved without neurological deficits by measurement of cerebral oxygen saturation and introduction of VA

ECMO. Nihon Kyukyu Igakukai Zasshi: Journal of Japanese Association for Acute Medicine. 2022;33(12):1028-32.

Nakahori Y, Hirose T, Shiozaki T, Ogawa Y, Ohnishi M, Fujimi S, et al. Serial changes in values of cerebral regional saturation of oxygen (rSO2) during resuscitation in patients with out-of-hospital cardiac arrest. Nihon Kyukyu Igakukai Zasshi. 2013;24:774-80.

Duplicates

Ito N, Nanto S, Nagao K, Hatanaka T, Nishiyama K, Kai T. Regional cerebral oxygen saturation on hospital arrival is a potential novel predictor of neurological outcomes at hospital discharge in patients with out-of-hospital cardiac arrest (vol 83 pg 46 2012). Resuscitation. 2014;85:1120.

Wesley K, Wesley K. CEREBRAL OXYGENATION. JEMS: Journal of Emergency Medical Services. 2015;40:25-.
